# Marine seafood production via intense exploitation and cultivation in China: Costs, benefits, and risks

**DOI:** 10.1371/journal.pone.0227106

**Published:** 2020-01-17

**Authors:** Cody Szuwalski, Xianshi Jin, Xiujuan Shan, Tyler Clavelle

**Affiliations:** 1 Alaska Fishery Science Center, National Oceanic and Atmospheric Administration, Seattle, WA, United States of America; 2 Chinese Academy of Fishery Sciences, Yellow Sea Fisheries Research Institute, Qingdao, China; 3 Laboratory for Marine Fisheries Science and Food Production Processes, Pilot National Laboratory for Marine Science and Technology (Qingdao), Qingdao, China; 4 Bren School of Environmental Science and Management, University of California, Santa Barbara, CA, United States of America; Australian Bureau of Agricultural and Resource Economics and Sciences, AUSTRALIA

## Abstract

Identifying strategies to maintain seafood supply is central to global food supply. China is the world’s largest producer of seafood and has used a variety of production methods in the ocean including domestic capture fisheries, aquaculture (both freshwater and marine), stock enhancement, artificial reef building, and distant water fisheries. Here we survey the outcomes of China’s marine seafood production strategies, with particular attention paid to the associated costs, benefits, and risks. Benefits identified include high production, low management costs, and high employment, but significant costs and risks were also identified. For example, a majority of fish in China’s catches are one year-old, ecosystem and catch composition has changed relative to the past, wild and farmed stocks can interact both negatively and positively, distant water fisheries are a potential source of conflict, and disease has caused crashes in mariculture farms. Reforming China’s wild capture fisheries management toward strategies used by developed nations would continue to shift the burden of production to aquaculture and could have negative social impacts due to differences in fishing fleet size and behavior, ecosystem structure, and markets. Consequently, China may need to develop novel management methods in reform efforts, rather than rely on examples from other large seafood producing countries. Improved accounting of production from fisheries and aquaculture, harmonization and centralization of historical data sets and systematic scientific surveys would improve the knowledge base for planning and evaluating future reform.

## Global seafood production

**Fig 1:**
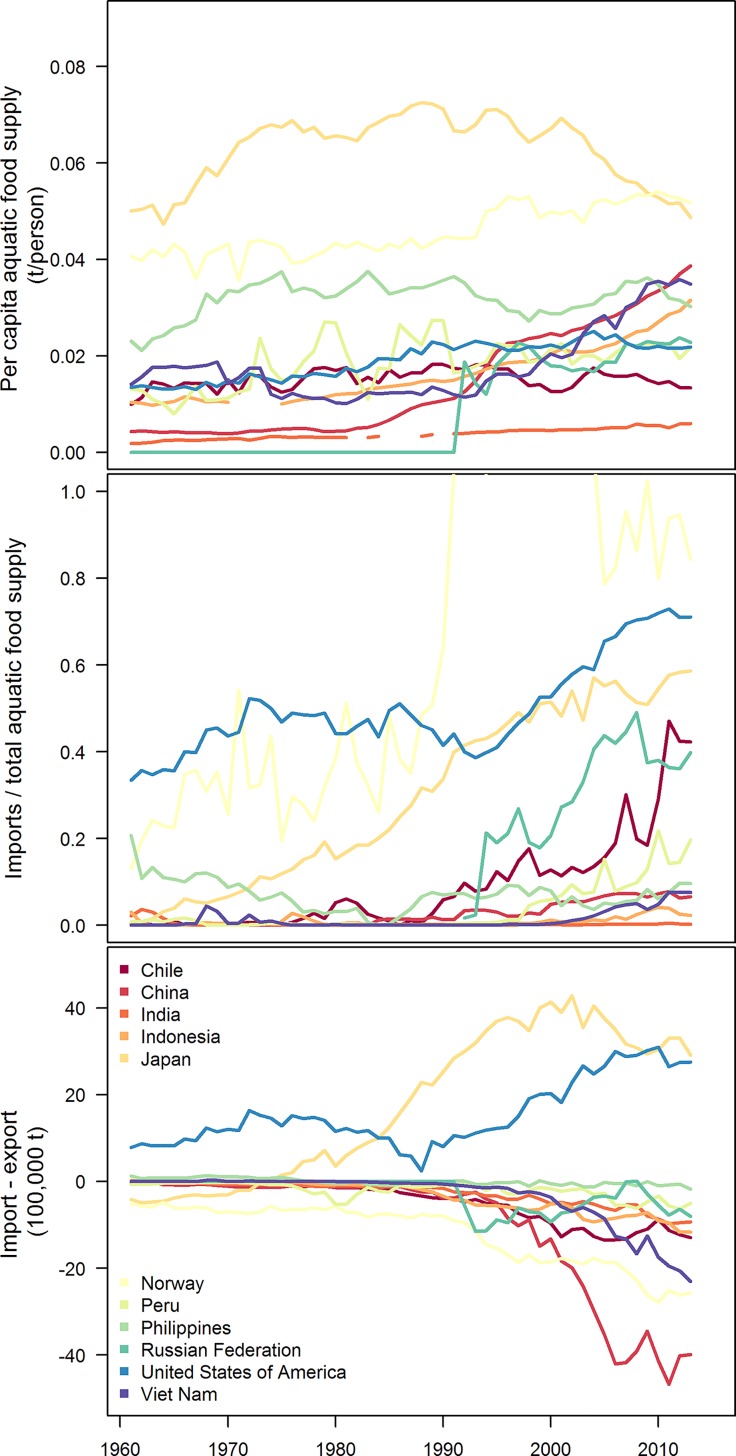
Seafood production metrics for the top 11 producing countries of seafood globally. Per capita aquatic food supply reported in tons (total production *minus* exports *plus* imports of aquatic food for human consumption; top), the percentage of total seafood supply made up by imports (middle), and the difference between imports and exports by country (color). Analysis based on FAO trade statistics (http://www.fao.org.fishery/statistics/software/fishstatj/en) and World Bank population data (https://data.worldbank.org).

**Fig 2:**
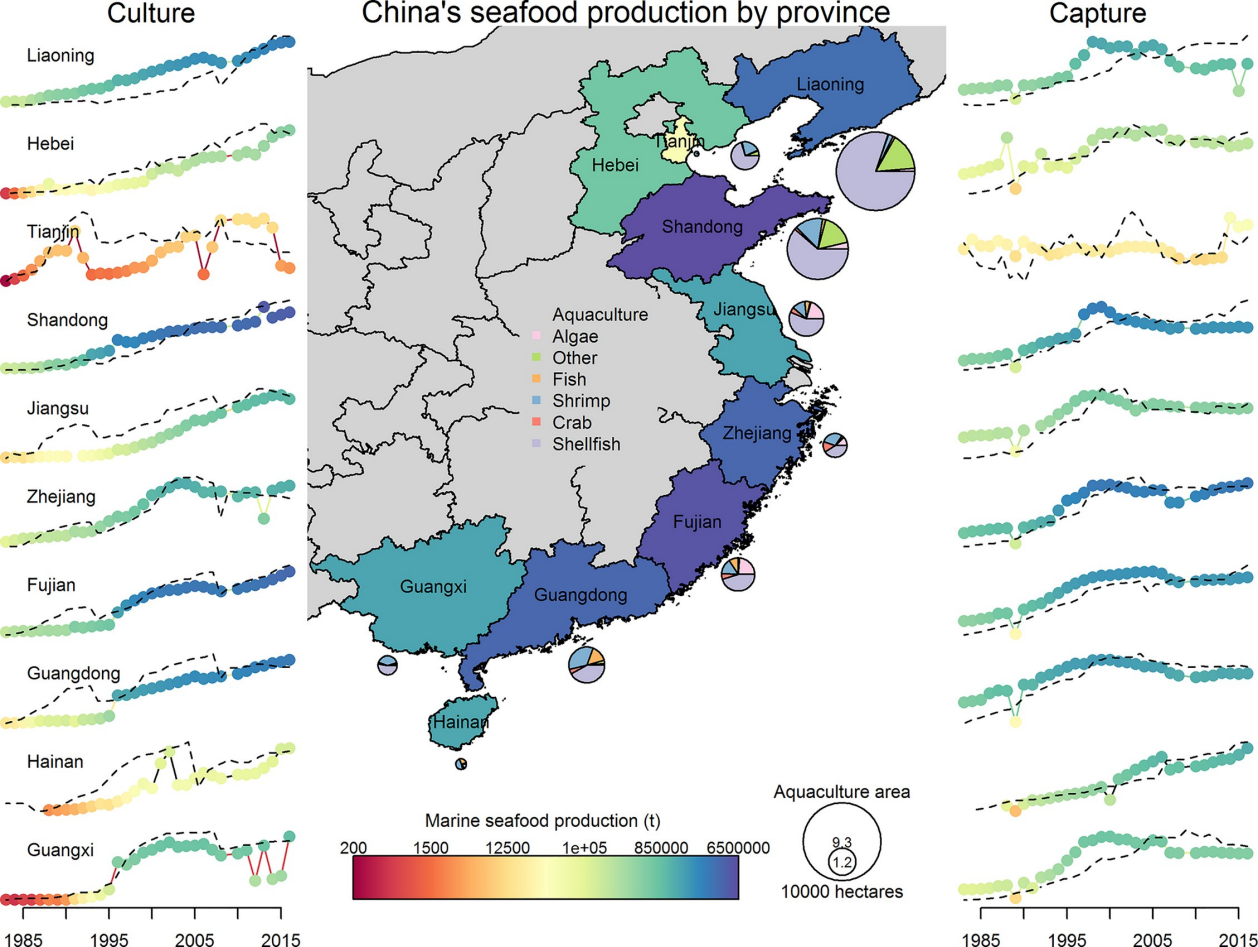
**Total marine production of cultured (left) and captured (right) seafood over time by province.** Data were not available for Chinese Taipei, so it was not included on the map. Dashed lines overlaid on culture and capture production by province represent the effort devoted to production (hectares and fleet power, respectively). Pie charts represent the relative size (not to scale) of area devoted to aquaculture in each province by product type. The magnitude of production by province is represented by color, with the scale indicated at the bottom of the figure.

Global seafood production reached 171 million metric tons in 2016, and on average ~17% of animal protein consumed globally comes from seafood (this number can be much higher for coastal communities; [1]). Marine capture fisheries contributed 79.3 million tons to production; marine aquaculture (mariculture) contributed 28.7 million tons. Demand for seafood globally is projected to increase as the population grows both in size and wealth [1] and identifying strategies of seafood production to satisfy this demand is a concern for nations reliant on ocean resources for protein and economic development. Approaches to seafood production vary across nations. Some countries, like the U.S.A., rely largely on capture fisheries to produce seafood (though a majority of consumed seafood in the U.S.A. is imported; Fig 1, [2]); other countries incorporate aquaculture more heavily to supplement fisheries (and sometimes far surpass fisheries, e.g. Vietnam; [1]). Fisheries management in many developed nations is focused on maintaining the productivity of individual species. In contrast, the fisheries in much of the developing world are often multi-species and minimally managed. Management of aquaculture is often reliant on the standards of the country in which the seafood is consumed, which varies globally [3].

China is the largest producer of seafood in the world, reporting 15.2 million tons of wild-capture marine fish (19% of global) and 49 million tons of cultured seafood (62% of global, including freshwater) in 2016 [1]. China has approached seafood production with multi-pronged strategies, including domestic wild capture fisheries, aquaculture, stock enhancement, distant water fisheries, and artificial reef building. China’s per capita aquatic food production increased dramatically through the 1980s and 1990s with the expansion of their aquaculture farms (both freshwater and marine) and improvement of their fishing fleets, reaching 38 kg per person in 2013—double the global average and 3^rd^ highest among the largest producing nations (Fig 1). The fraction of China’s seafood supply that is imported is small compared to other countries (~5% in 2013, 3^rd^ lowest among largest producers; Fig 1). Furthermore, China’s aquatic food trade balance is tipped heavily in favor of exports—they ran a trade surplus of ~4 million tons in 2013, which is largest export-shifted trade gap globally (Fig 1). By these measures, China is one of the most seafood secure (in terms of volume) of the largest seafood producing nations.

Our goal in this paper is to examine the costs, benefits, and risks of China’s seafood production strategies built around intense exploitation and cultivation of the ocean. We examine variation in returns of different product types for fishing effort and cultivated area across provinces and the interactions between wild and farmed systems. Currently, China is planning large-scale fisheries management reform and quota-based domestic fisheries management focused on single species is being discussed as a potential reform direction [4]. We conclude by discussing the potential impacts of reforming seafood production strategies towards those used in developed nations on China’s seafood production.

## Methods

We identify differences in the trends in production over time by species, group, and production mode using hierarchical clustering algorithms and multivariate regression. Product- and province-specific relationships between effort (either fishing effort or cultured area) and product yield were delineated using generalized additive models. We also examine the relationship between fisheries and the extent and intensity of mariculture efforts using generalized additive models.

### Data

Our primary source of data for fisheries and mariculture related quantities is the Chinese Fishery Statistics Yearbook [5]. The Fishery Statistics Yearbook data are collected via reporting forms filled out by enumerators at the village/town level (>40,000 individuals participate at the lowest level), which are compiled to the county/city/province level [6]. Once compiled, the data are entered into a database at the Bureau of Fisheries at the Ministry of Agriculture and published annually. In the fishing sector, medium to large-sized fishing vessels are required by regulation to record their catch on log sheets and submit them to the local agency. Data on small-scale fishing are collected by enumerators at the village level and sent upward to the county level. Aquaculture production data are also collected by enumerators at the village level; data on aquaculture extent is collected from paperwork required for obtaining a license to establish or modify an aquaculture facility. Data on fishing vessels within the Fishery Statistics Yearbooks are obtained from vessel registration records. We include a comparative analysis using the reconstructed fisheries catches from the Sea Around Us (SAU) database to explore the impact of different data sources for fisheries catch on the inference on optimal effort levels [7].

### Production over time

Understanding changes in seafood production over time is a central question in attempting to understand the costs and benefits of a seafood production strategy. Here, we identified differences in production by product and production mode in China using hierarchical clustering of production time series (i.e. biomass of caught or cultivated species or species groups) and multinomial logistic regression. Clustering was performed at the national level and within each province. Time series of production for the provincial clustering were only included in the analysis if its mean exceeded 15,000 tons (n = 164). Time series were scaled to have a mean 0 and standard deviation 1 before clustering so that the ‘shape’ of the time series determined cluster membership. Four clusters of production time series were identified in both the national totals and provincial data: increasing, plateaued, dome-shaped, and decreasing. Hierarchical clustering was implemented on a Euclidean distance matrix derived from the production time series using *hclust* in the *stats* R package [8]. Membership within a cluster was predicted via multinomial logistic regression fitted using neural nets in *multinom* in the R package *nnet* [9]. Multinomial logistic regression predicts the log odds of a given outcome as a linear function of predictors, given a ‘reference’ scenario (‘decreasing’ catch was chosen arbitrarily as the reference here). For example, the log odds of a time series of fisheries catch belonging to a given cluster relative to a baseline cluster can be represented as:

$$\ln\left(\frac{p(Y_{i}=j)}{p(Y_{i}=ref)}\right)=\alpha_{j}+\beta_{1}P_{i}+\beta_{2,i}R_{i}+\beta_{3,i}E_{i}$$

Where *p(Y*_*i*_ *= j)* is the probability of membership of observation *Y*_*i*_ in production cluster *j*; *p(Y*_*i*_ *= ref)* is the probability of membership in the ‘reference’ production cluster, α_j_ is a production cluster specific intercept, and β_1–3_ are estimated coefficients determining relationship between the probability of observing production cluster *j* given categorical variables. The categorical variables used as predictors here were: product type (P; crustacean, fish, shellfish, invertebrate, algae), production mode (R; cultivated or farmed), and effort trajectory (E; increasing, decreasing, plateau, dome-shaped). Clusters of effort trajectories were identified in the same manner as production time series above (S11 Fig).

### Returns on effort by province and product

The relationship between cultured and captured production (*P*_*p*,*y*_) and effort (cultivated hectares and fleet power, respectively; *E*_*p*,*y*_) for 4 product types were estimated by generalized additive models (GAMs), assuming Gaussian error and using thin-plate splines as the basis [10]. The number of knots and smoothing parameters are estimated within the model fitting via generalized cross validation.

$$P_{p,y}=\alpha +s(E_{p,y})+\varepsilon_{p,y};\;\varepsilon ∼N(0,\sigma_{g})$$

GAMs were used rather than traditional production models sometimes used in fisheries science because trophic dynamics of the ecosystems have changed over time, which violates the assumptions of production models. We report the deviance explained and the significance of the smooth term by province. The fits of GAMs to both Sea Around US data and Chinese Yearbook data are compared.

### Interactions between mariculture and fisheries

GAMs were also used to estimate relationships between the extent of mariculture (A_p,y_; crustacean, shellfish, fish, algae) and the total fisheries catch (in tons) per unit effort (kilowatts) by province and year (CPUE_p,y_). Total stock enhancement in numbers of individuals by product type (fish and crustaceans; E_p,y_) was also included as a covariate to predict total fisheries CPUE.

$${CPUE}_{p,y}=\alpha +s(A_{p,y})+s(E_{p,y})+\varepsilon_{p,y};\;\varepsilon ∼N(0,\sigma_{s})$$

GAMs are useful here because they can capture non-linear relationships between variables and it is unclear what relationships should be expected between mariculture and fisheries *a priori*, given the range of observations in the literature. The number of knots permitted in this section of the analysis was limited to 3, given the small sample sizes. If the number of knots available to the model for each form of aquaculture was not constrained, the models did not converge.

GAMs were also used to examine the relationship between total fisheries CPUE in a province *r* in year *y* (*CPUE*_*r*,*y*_) and the extent and intensity (cultivated biomass per hectare) of mariculture in province *r* during year *y* of product type *p* (A_r,y,p_ and I_r,y,p_, respectively). Intensity and extent of mariculture were scaled within a province and a two-dimensional smoothing term was estimated in the GAM relating total fisheries CPUE to different types of mariculture (fish, crustacean, algae, and shellfish). Province (R) was included as categorical predictor.

$${CPUE}_{r,y}=\alpha +s(A_{r,y,p},I_{r,y,p})+\beta_{r}R+\varepsilon_{y};\;\varepsilon ∼N(0,\sigma_{r})$$

CPUE was calculated as the catch (e.g. total fisheries catch or catch of shellfish in Zhejiang province) divided by the total provincial fisheries effort in terms of the fleet power in kilowatts. The number of knots allowed in the smooth for this portion of the analysis was estimated within the fitting of the GAM for all product types, except for algae, which limited to 7 given concerns of overfitting.

## Results

### Domestic marine capture fisheries

**Fig 3:**
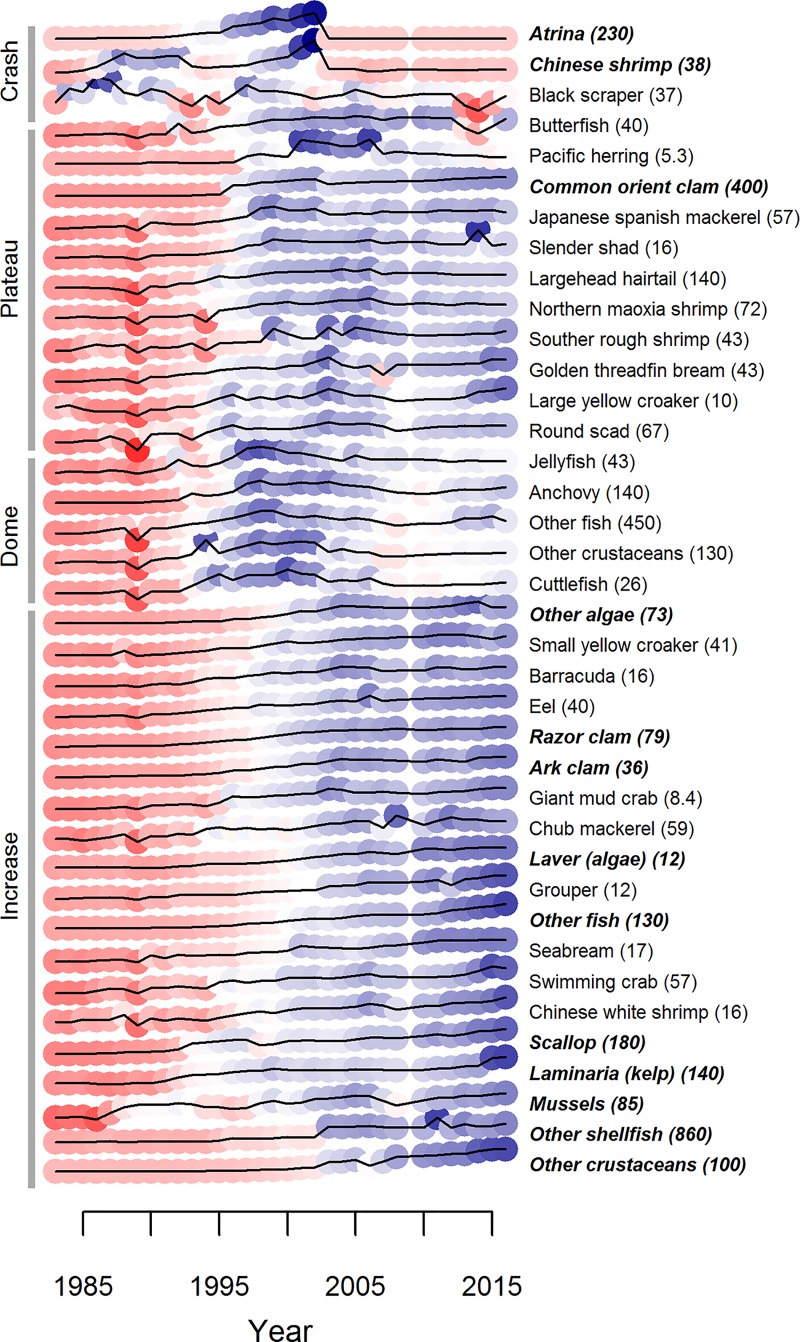
National totals for marine seafood production by species/groups from 1983–2016. Time series are grouped via hierarchical clustering so that time series with similar ‘shapes’ are together. Colors represent the value of a species in a given year relative to the arithmetic mean for that species/group. Red values are below the mean; blue values are above. Names printed in bold italics are aquaculture production and the number in parentheses is the maximum value for that species/group in 10,000 t.

**Fig 4:**
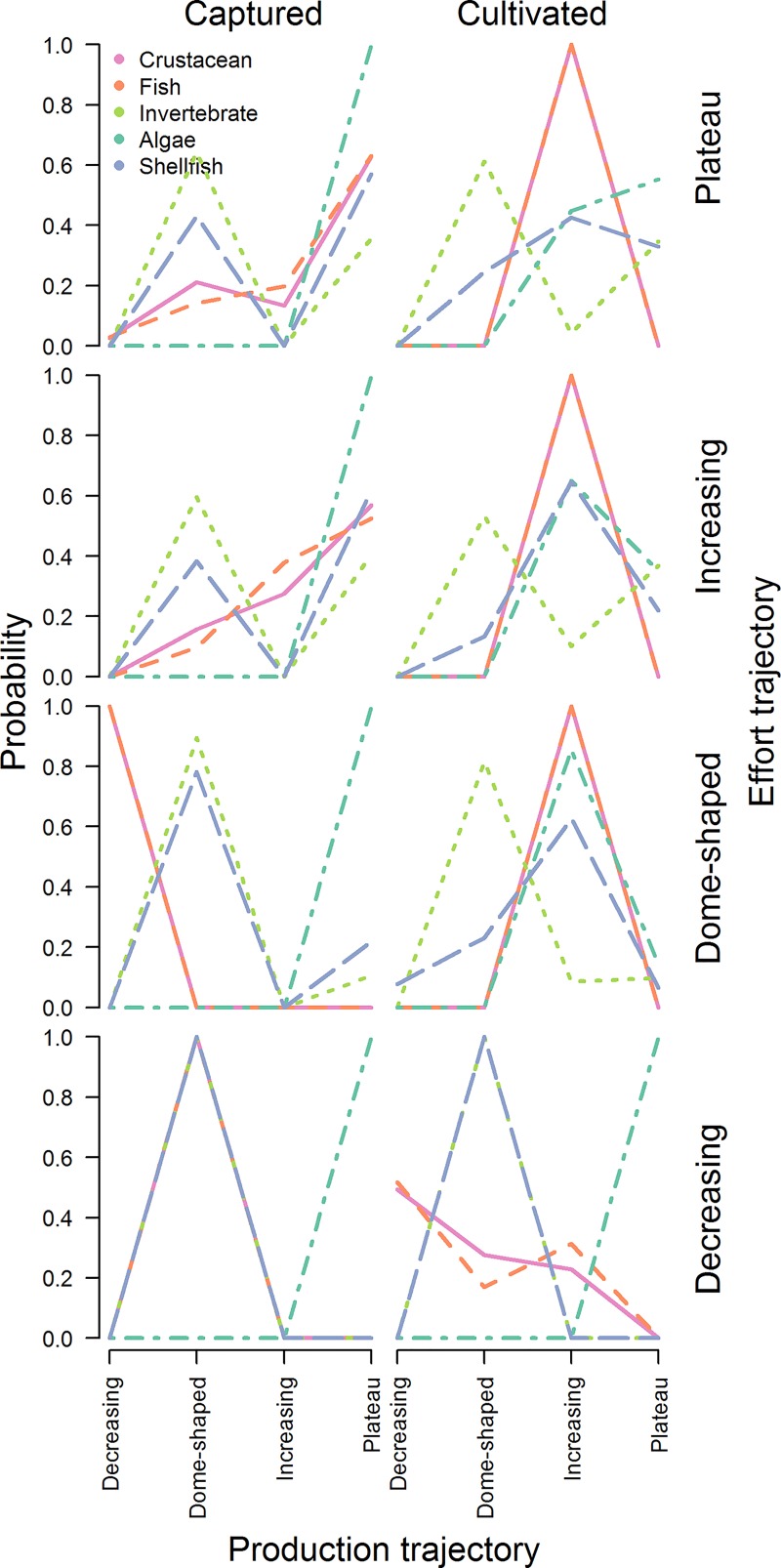
Probabilities of a production trajectory given production mode, product, and effort trajectory estimated via multinomial logistic regression. Probabilities sum to one for a given effort trajectory, product type, and production mode.

**Fig 5:**
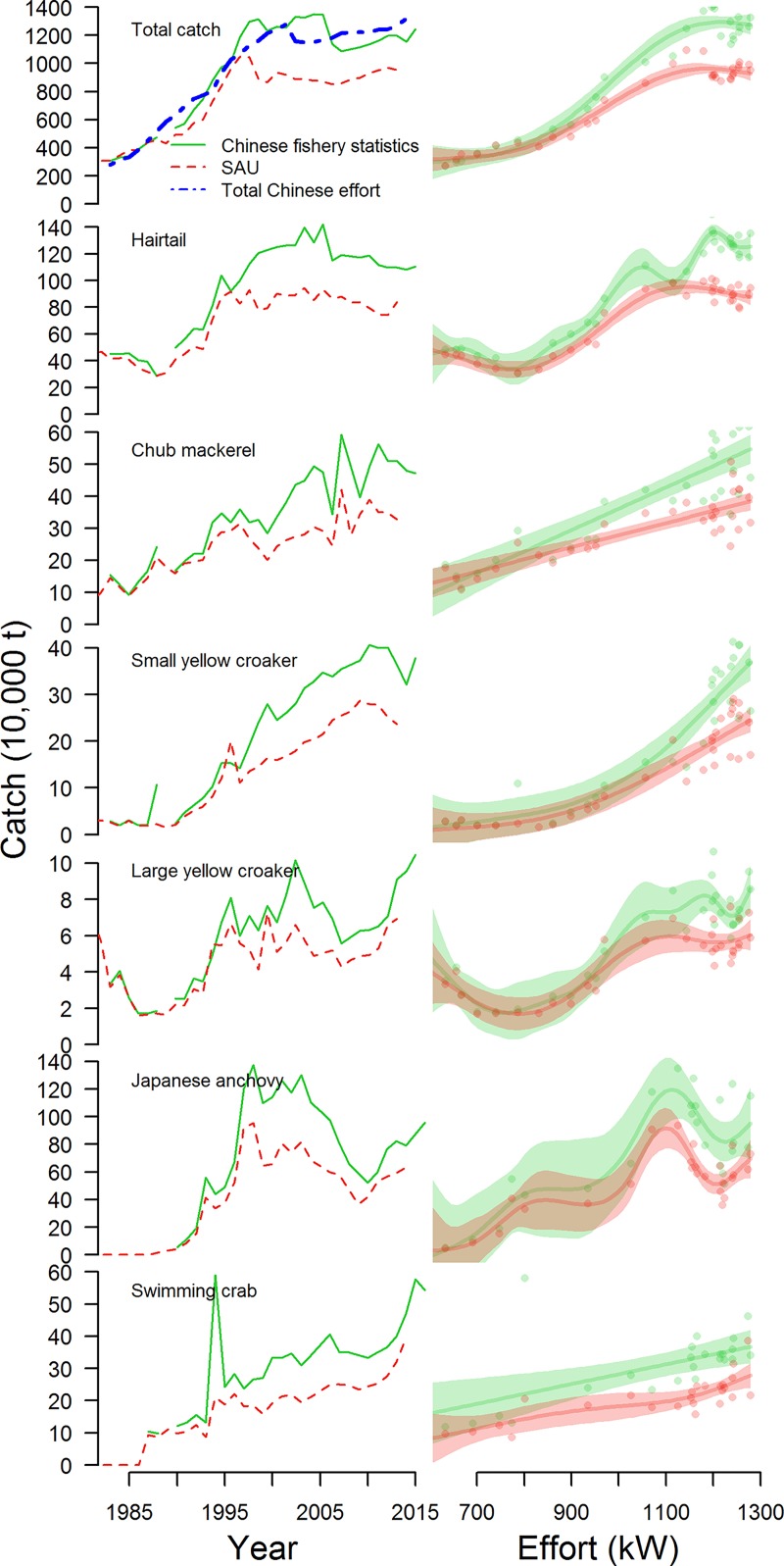
A comparison of reported Chinese fisheries catches from the Chinese fishery statistical yearbooks and the sea around us reconstructions for the Chinese EEZ. Total fishery catches are compared, in addition to 6 important commercial species. Right column is the fit of a GAM that estimates catches from effort for both the SAU data [7] and the Chinese data [5]. Effort is the total reported power of the Chinese domestic fleet in kilowatts [5].

**Fig 6:**
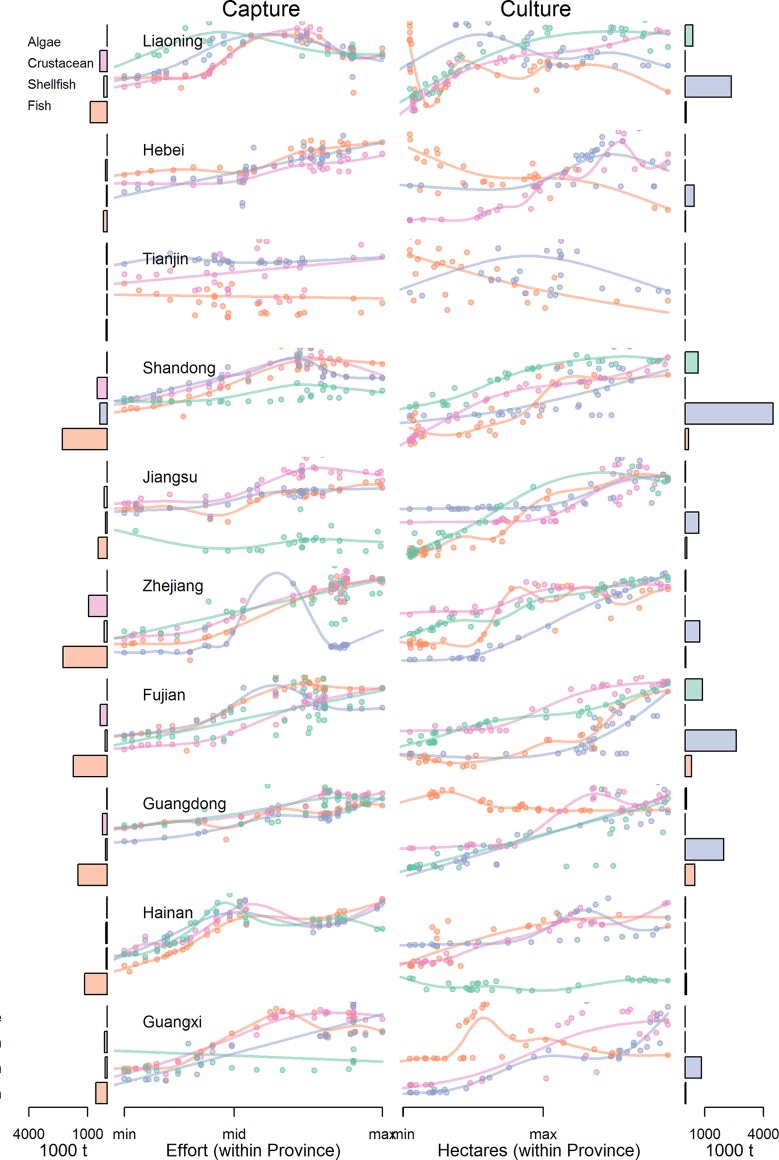
Production (captured or cultured) vs. input (effort or area) by province and product for domestic production. Effort and area are scaled within a province to improve comparability. Barplots on the edges of Figs represent the maximum production by product type from 1983–2016 for a given province. Fitted relationships between effort and production are estimated with GAMs.

**Fig 7:**
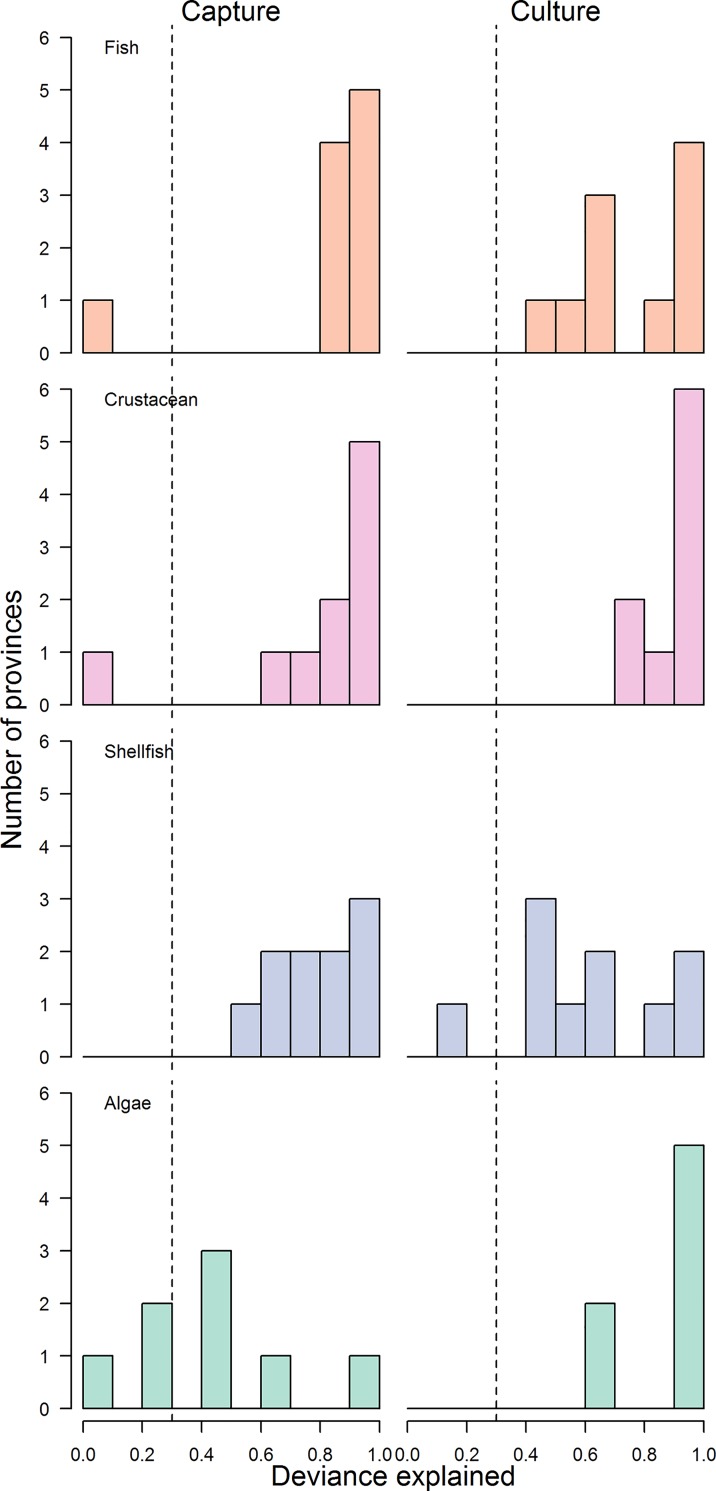
Histograms of the deviance explained from GAMs relating production and effort for captured and cultured products. Dotted vertical line represents the level of deviance explained beneath which effort was not a significant predictor of production within the GAMs.

China’s fishing fleets fish primarily with indiscriminate gear like trawl and gill nets [5]. Fishing effort (as seen through fleet power) has increased continually since the 1980s for five of China’s coastal provinces, with the other provinces plateauing in the early 2000s (Fig 2). Total fishery catches plateaued in the late 1990s for 9 of 10 provinces, but the response of individual species at both the national and provincial level to changes in effort has varied (Fig 3; S1–S10 Figs for provincial level). Across provinces, crustaceans and fish were significantly more likely to have increased continually over time, whereas other invertebrates and shellfish were more likely to have dome-shaped production trajectories (Fig 4; Table 1). In general, captured products were significantly more likely to have dome-shaped or decreasing trajectories than cultivated products (Table 1). At the national level, effort was a significant predictor of catch (p < 0.01) and the total catch returned for a given amount of effort is currently higher than that which produced the maximum observed catch (Fig 5). GAMs fit to the SAU data all reported effort as a significant predictor of catch and produced smooth relationships between catch and effort with very similar shapes to those fit to the reported fisheries catches. The key difference between the relationships was the scale; the inflection points in yield (if they existed) appeared at similar levels of effort for both data sets. The catch returned for a given harvest effort varied by province and product type (Fig 6). Effort was a significant predictor of production by product in all cases except 5, with 4 of those cases coming from capture fisheries (Fig 7). The deviance explained by the GAMs was generally higher for aquaculture than fisheries. In many provinces, higher catches were associated with less fishing effort than is currently expended, but some provinces still report linear increases in catch for increases in effort.

**Table 1:** Predicting trajectory of production by factors (product type, production mode, effort, and the interaction between production mode and effort). Reported numbers are the coefficient associated with a given trajectory for a given factor, relative to the reference trajectory (‘decreasing’) and factor (e.g. product types are relative to ‘algae’). Coefficients can be interpreted to represent the magnitude and direction of the effect of a given category relative to the reference. For example, crustaceans are significantly more likely to have increasing or plateauing production trajectories than algae, when controlling for other factors. Numbers in parentheses are the standard errors of the estimated coefficient. Standard errors are not shown when the sample size for a given combination is too small to calculate them. Asterisks indicate significant effects.

	*Trajectory of production*		
	Dome-shaped	Increasing	Plateau
Crustacean	-0.695 (-0.738)	10.565*** (-0.772)	-30.425*** (-0.781)
Fish	-1.228 (-0.81)	10.828*** (-0.857)	-30.560*** (-0.656)
Invert	21.774*** (-0.33)	-8.156 (-9.685***)	-0.33
Shellfish	21.783*** (-0.569)	-12.156*** (-0.54)	-12.641*** (-0.613)
Aquaculture	-9.898*** (-0.922)	3.511*** (-0.84)	-23.348*** (-0.41)
Dome-shaped effort	-10.400*** (-0.495)	12.793*** (-0.516)	10.631*** (-0.417)
Increasing effort	17.802*** (-0.417)	32.051*** (-0.403)	44.471*** (-0.332)
Plateau effort	-7.188*** (-0.766)	5.950*** (-0.801)	19.029*** (-0.444)
***Interactions***			
Dome-shaped cultivation effort	-10.400*** (-0.495)	12.793*** (-0.516)	10.631*** (-0.417)
Increasing cultivation effort	-9.891*** (-0.463)	22.677*** (-0.372)	6.899*** (-0.386)
Plateaued cultivation effort	0.593 (-0.554)	33.679*** (-0.443)	18.683*** (-0.438)
Constant	10.013*** (-0.313)	-14.844*** (-0.293)	14.570*** (-0.319)

*p<0.1

**p<0.05

***p<0.01

Total catches of some species show an increasing trend from the early 1980s to present (e.g. small yellow croaker (*Larimichthys polyactis*) and chub mackerel (*Scomber japonicus*); Fig 3), while others began to decline in the late 1990s-early 2000s (e.g. largehead hairtail (*Trichiurus lepturus*) and cuttlefish (*Sepiina spp*.); Fig 3). A large fraction of the catch (~40%) is not classified to species or genus and includes both larger fish and ‘trash’ fish (i.e. fish not used for human consumption). Catch for this ‘other’ category has declined 33% since its peak in 1998, presumably due to both changes in the number of species included in the ‘other’ category (some new species started to be enumerated in the 2000s) and changes in the numbers of ‘other’ fish in the ocean. Total fish catches are generally less sensitive to effort over the range of observed effort, while crustacean catches display larger declines or plateau earlier than fish. Provinces like Liaoning and Guangxi have reached fleet powers at which diminishing returns are apparent, yet returns for other provinces like Zhejiang and Jiangsu have yet to level off or decrease.

Zhejiang, Shandong, and Fujian Province are the largest fishing provinces in China, reporting 3.2, 2.1, and 1.9 million tons of wild-capture seafood in 2016, respectively. For reference, Indonesia, the United States, and Russia (the countries ranked 2–4 in terms of marine capture production behind China) produced 6.0, 4.9, and 4.0 in 2014, respectively [1]. As of 2016, Zhejiang’s fleet is the most powerful of China’s provinces, with 19,493 vessels reporting a fleet power of 3,666,831 kilowatts. Fujian’s fleet is the most numerous with a fleet of 30,680 vessels at 2,235,036 kilowatts and Shandong follows with 25,081 vessels at 1,824,157 kilowatts.

Upwards of 95% of reported catches by Zhejiang fleets are captured in the East China Sea. Trends in catches of large yellow croaker (*Larimichthys crocea*) and black scraper (*Thamnaconus modestus*) have continually declined since 1983 (S1 Fig). Trends in catches of ‘other fish’, cuttlefish, jellyfish (*Rhizostomatidae*), ‘other crustaceans’, and largehead hairtail are dome-shaped with the most recent years leveling off. Catches for all other species/groups have continually increased over the period of the data, following changes in fleet power (Fig 2). Currently, largehead hairtail, ‘other crustaceans’, northern maoxia shrimp (*Acetes chinensis*), and ‘other fish’ comprise nearly two thirds of the catch of Zhejiang’s fleets. Effort has yet to reach a level at which yield declines for algae, fish, and crustaceans; yield of shellfish is markedly dome-shaped (Fig 6).

Shandong’s fleets fish primarily in the Yellow Sea and the Bohai Sea. The trends in catch time series from Shandong’s fleets can be classified into ‘declining’, ‘dome-shaped’, and ‘plateaued’ (S2 Fig). No species/group has continually increased over the period for which data are available. Black scraper, cuttlefish, Chinese white shrimp (*Fenneropenaeus chinensis*), and slender shad (Pristigasteridae) have decreased continually since 1983. Some dome-shaped time series are very punctuated, with high catches only lasting several years (e.g. pacific herring (*Clupea pallasii*), giant mud crab (*Scylla serrata*)); others have ‘domes’ stretching over decades (e.g. jellyfish and anchovy (*Engraulis japonicus*)). Anchovy, ‘other fish’, ‘other shellfish’, and Japanese Spanish mackerel (*Scomberomorus niphonius*) comprise >70% of Shandong’s catch. Observed yield curves are more strongly dome-shaped for shellfish and crustaceans than for fish or algae (Fig 6).

Catches for nearly all species and groups from Fujian province have continually increased over time or plateaued in the late 1990s (S3 Fig). Large and small yellow croaker are exceptions to this rule. The composition of catch is more diverse in Fujian, perhaps because ~10% of catch comes from the South China Sea and the fleets likely operate in more southern waters of the East China Sea (from which the remaining 90% of their reported catches are derived) when compared to Zhejiang. “Other fish”, round scad (*Decapterus punctatus*), largehead hairtail, and chub mackerel (*Scomber japonicus*) comprise only 50% of the catch, which is less than the top four species/groups from Zhejiang and Shandong which comprise >70% of reported catch. Observed yield curves are dome-shaped for fish and shellfish in Fujian; yield for algae and crustaceans continues to increase over the observed range of effort (Fig 6).

### Distant water fisheries production

**Fig 8:**
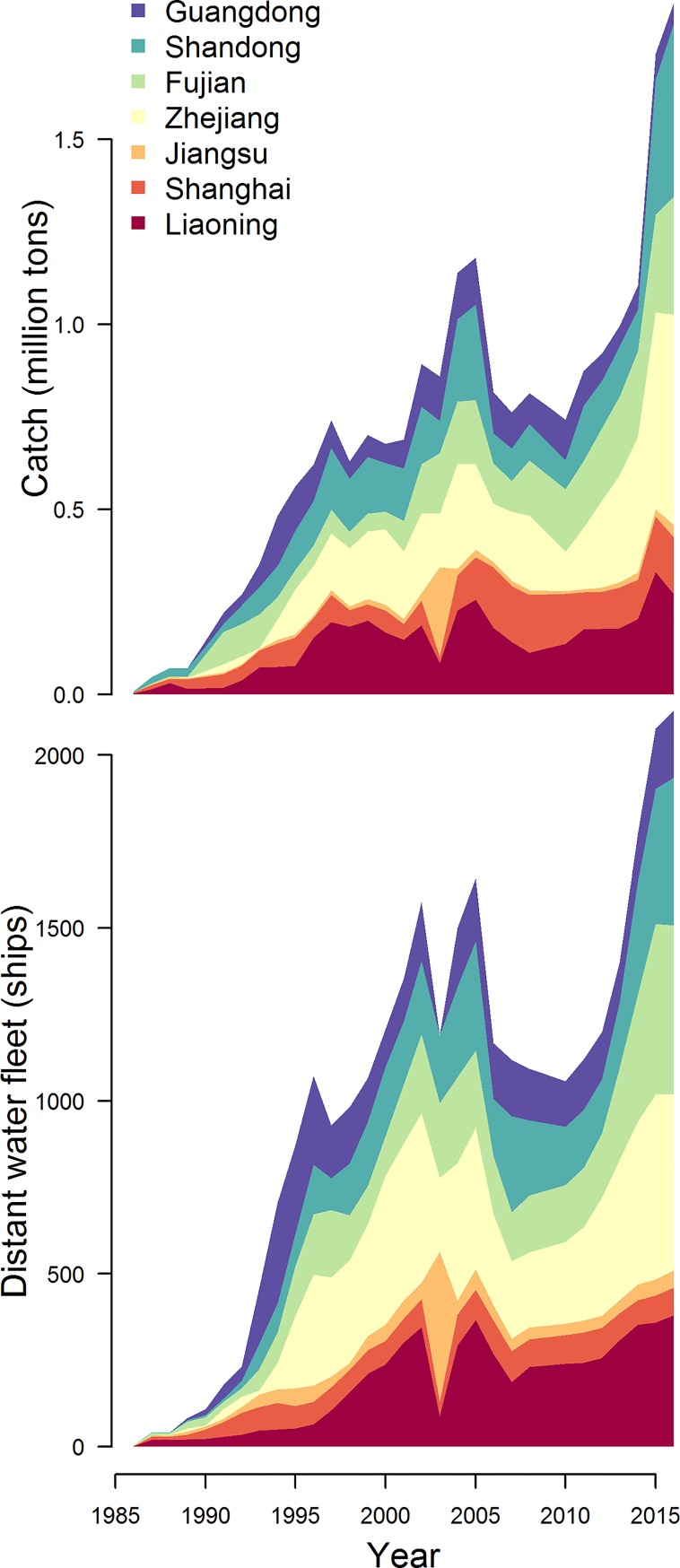
Catches from distant water fisheries and distant water fleet size by province for the 7 largest producing provinces.

China’s distant water fisheries began in the mid-1980s and have grown to ~1.9 million tons reported in 2016 (Fig 8, [5]). Approximately 2,180 Chinese vessels participated in the distant water fisheries in 2016 (Fig 8). Zhejiang’s distant water fleet is the most productive in China, with a capture production of ~680,000 tons in 2016; Shandong has the second most productive distant water fleet, with a capture production of ~460,000 tons in 2016 (Fig 8). In a global context, reported catches from China’s distant water fleet placed it as the 10^th^ largest producer of capture seafood in 2016. The dominant gears in distant water fisheries include trawlers, purse seiners, and long liners. The waters from which China’s distant water catches are taken are not reported in the Fisheries Yearbooks, but others have reported that Chinese vessels have been observed fishing in the EEZs of 93 maritime countries [11]. In these same reports, estimates of China’s distant water catches were placed closer to 4.6 million tons based on Monte Carlo simulations incorporating observations of 500 records of Chinese vessels fishing in distant waters from 2000 to 2011. However, the distributions used in [11] appear to overestimate of catches by gear type given the reference data sets (Fig 3 in [11]). The distribution for bottom trawlers is particularly concerning given that the estimated 345 bottom trawlers in West Africa contributed 2.9 million tons to Pauly et al.’s [11] distant water catch estimates. Some size classes of catch from the distribution used to estimate catches from bottom trawlers (e.g. the mid-point of the 32–100 ton bin) exceed the observed data by >100% (Fig 3 in [11]). China’s distant water fleets are a contentious topic and it is uncertain how to best approach understanding their dynamics given the private nature of fishing agreements made with other countries.

### Cultured seafood

**Fig 9:**
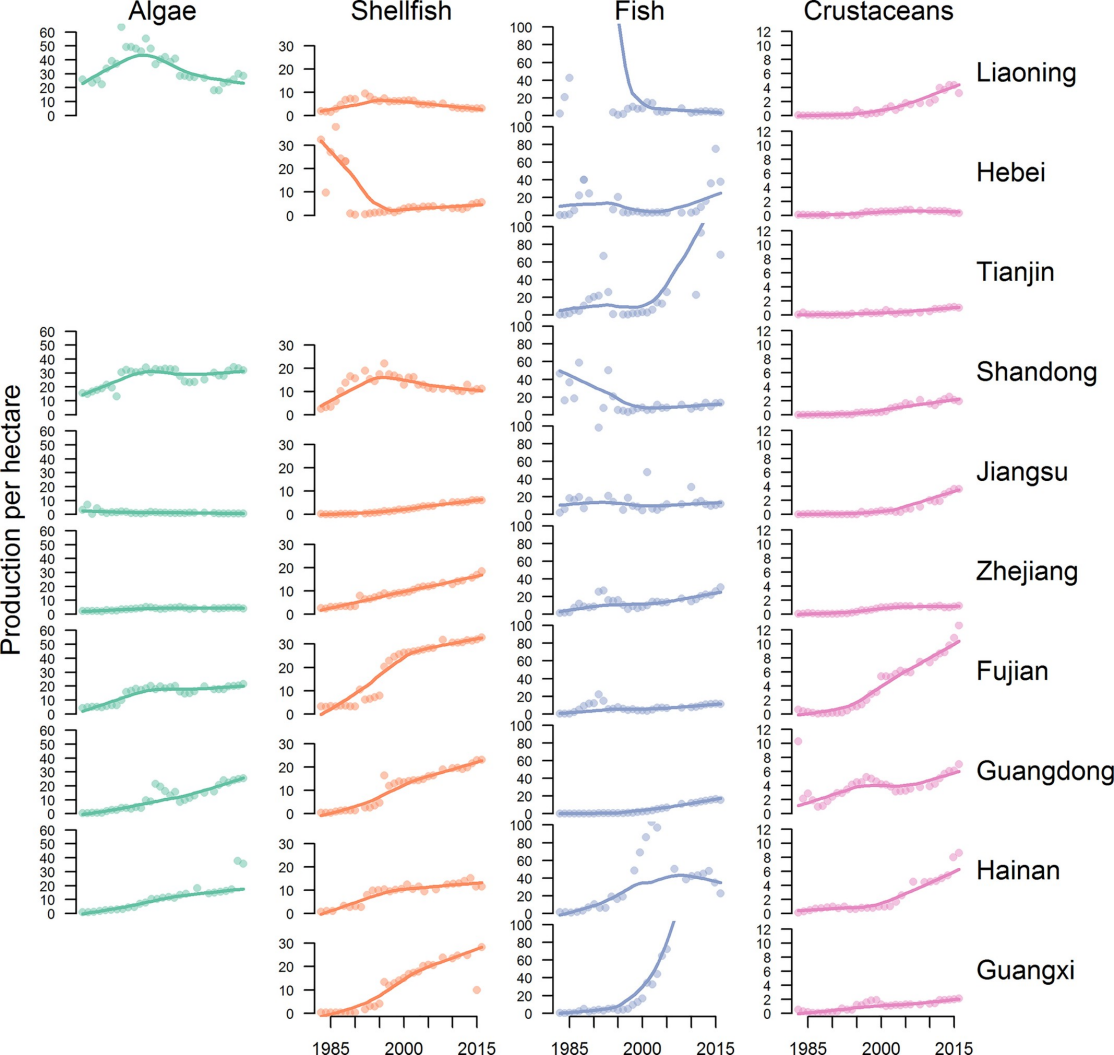
Production per hectare of mariculture products over time by province with LOESS line overlaid.

China’s mariculture production surpassed fisheries production in 2006 and remains the country’s fastest growing food sector (5–6% per year). Across provinces, time series of farmed products are significantly more likely than captured seafood to have continually increased over time (Table 1). National mariculture policy has shifted from increasing output via the expansion of farming areas to increasing production and quality per unit area through technological innovation (intensification) and integrated farming practices [12] (Fig 9). In total, cultured area has expanded from ~186,000 hectares to 2.3 million hectares over the period 1983 to 2015 while production per hectare has increased for most product types in most provinces, presumably resulting from improved farming practices [13] (Fig 9).

However, environmental degradation arising from an early focus on intensive monoculture led other species to decline or disappear completely (e.g. Chinese shrimp, *Penaeus orientalis* [14]). In response, China’s mariculture strategy has shifted towards polyculture systems, where fed species (fish and crustaceans) are farmed in close proximity to non-fed species (bivalves and seaweeds) that filter and reduce waste [13]. China has a long history of polyculture in freshwater [15]) and there are notable marine examples (e.g. Sanggou Bay [16]; Zhangzidao [17]). Still, it will take time to increase the prevalence of polycultures in the marine environment given the inertia of existing systems. Diminishing returns on the area devoted to mariculture are evident, but the point at which these diminishing returns are reached varies by type of mariculture and province (Fig 6). Several explanations for diminishing returns are plausible, including saturation of suitable habitat, disease, density dependent issues like growth limitations, and access to high-quality feed.

Shandong, Fujian, and Guangdong provinces are the largest producers of mariculture products in China with 5.0, 4.3, and 3.0 million tons produced in 2016, respectively. For reference, North and South America combined produced 2.2 million tons, Europe produced 2.5 million tons, and Asia in total produced 21.8 million tons in 2014 [1]. Shandong devoted 560,000 hectares to mariculture in 2016, Fujian devoted 166,000 hectares, and Guangdong utilized 195,00 hectares.

Shellfish production in Shandong has increased continually over time and makes up a large fraction of total production—common orient clam (*Meretrix lusoria*), ‘other shellfish’, scallops (*Pectinidae*), and mussels (*Mytilidae*) made up >76% of the mariculture product in 2016. Fish mariculture has also increased continually over time, but only made up 3% of mariculture production in 2016. Production of Chinese white shrimp and atrina (*Pinnidae*) declined sharply in the late 1990s and have not increased since (S2 Fig). Intensity (as seen through production per hectare) has increased for shellfish in Shandong, but has declined for crustaceans, algae, and fish since the late 1990s (S12 Fig). The area devoted to culture of all products has increased over time, but shellfish has plateaued in recent years (Fig 9).

Shellfish makes up a smaller fraction of the mariculture portfolio in Fujian province than Shandong, but is still the most produced product. The top four products in 2016 were ‘other shellfish (49%), luminaria (*Laminariaceae*; 15%), common orient clam (8%), and ‘other fish’ (8%), comprising 80% of total production. Algae plays a larger role in Fujian’s production than in other provinces, comprising 16% of the total in 2016. As in Shandong, production of Chinese white shrimp and atrina declined sharply in the late 1990s, but, in contrast to Shandong, common orient clam also declined (S3 Fig). The intensity of culture has increased for all product types in Fujian, which reports the highest shellfish and crustacean production per hectare of any province (Fig 9). The area devoted to all products has increased over time, but algae in particular displays an exponential increase (Fig 9).

The top four mariculture products in Guangdong in 2016 were ‘other shellfish’ (46%), ‘other fish’ (16%), ‘other crustaceans’ (15%) and common orient clam (11%), comprising 88% of the total mariculture production (S5 Fig). Crustaceans play a larger role in Guangdong’s production portfolio than the other top producing provinces. Chinese shrimp and atrina production also declined in the late 1990s in Guangdong; common orient clam also declined, but not to the extent it did in Fujian. The intensity of culture in Guangdong has continually increased, but has not yet reached the levels seen in Fujian (Fig 9). The area devoted to algae and shellfish has increased over time in Guangdong, but area devoted to fish culture has continually decreased and crustacean area plateaued in the early 2000s (Fig 9).

### Mariculture interactions with fisheries

The effects of mariculture on wild capture fisheries are diverse [18]. Farms can attract wild fish by providing structure and food [19], and farms may further change the make-up of fish communities near the farm by altering habitat [20] or impacts on recruitment [21]. Mariculture and hatchery releases of fish have been implicated in increased disease outbreaks and decreased genetic diversity among wild populations [22]. In China, past outbreaks of disease have cost tens of billions of dollars [23]. Shrimp farming has been implicated in the destruction of essential fish habitat in mangroves and, together with finfish mariculture, increased pollution in coastal waters [23]. Although added nutrients can degrade habitat and water quality, they may increase fishery landings and biodiversity in some cases [24].

**Fig 10:**
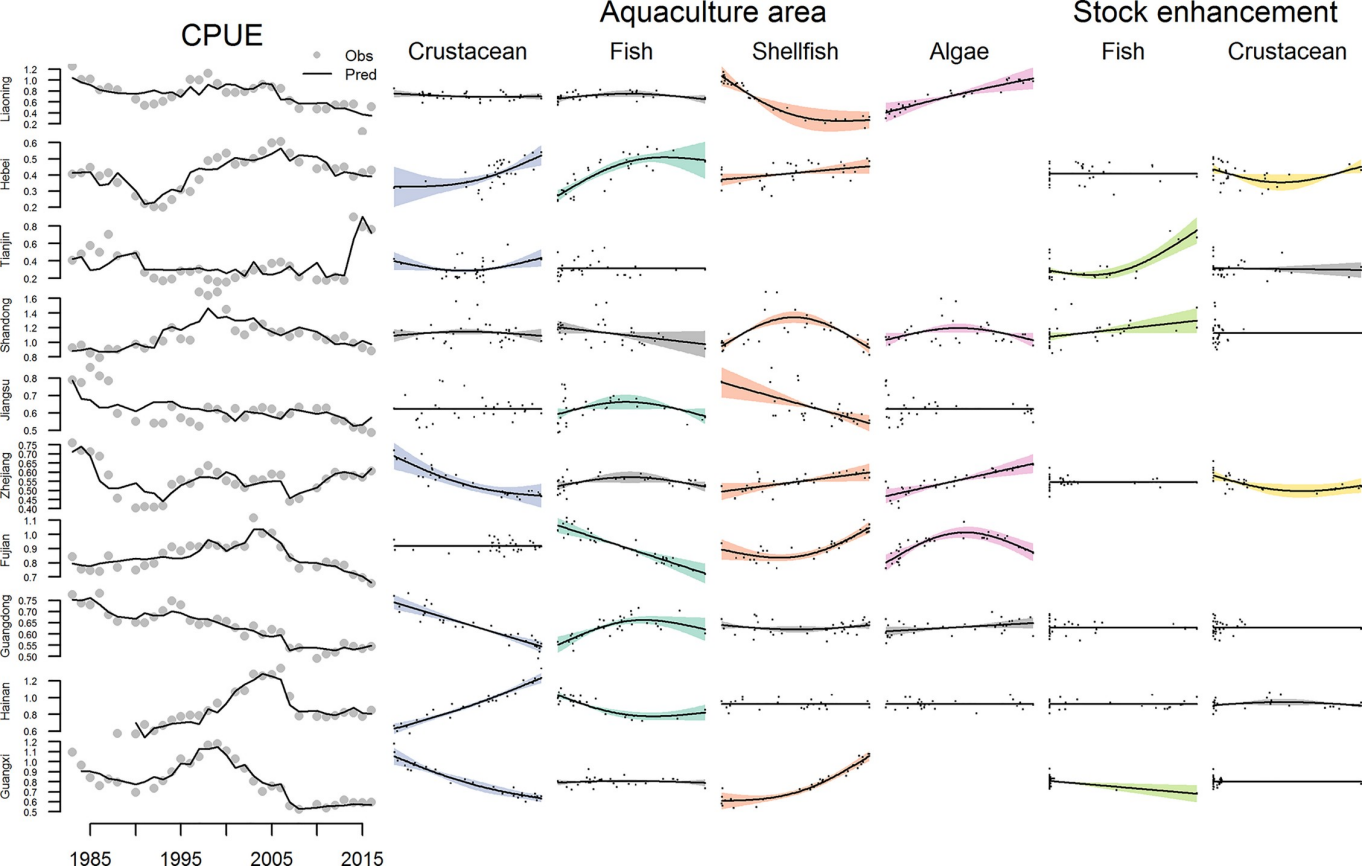
**Fits (black lines; left column) of GAMs predicting province level catch per unit effort data (grey dots; left column) by the area of aquaculture by product and the amount of stock enhancement by product.** Plots of the relationships between aquaculture area or stock enhancement and CPUE in color indicate a significant relationship (p < = 0.05).

**Fig 11:**
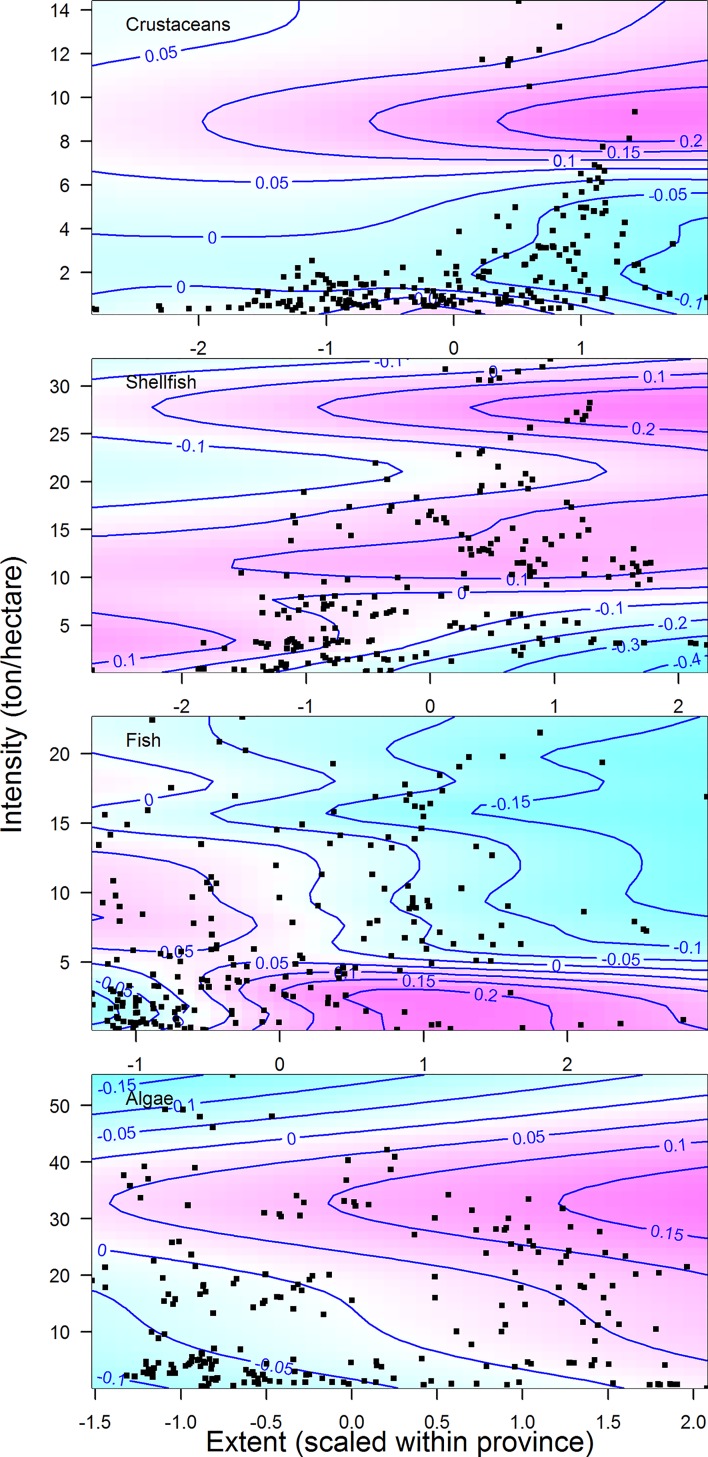
Two-dimensional GAMs relating total fisheries CPUE and the intensity and extent of different aquaculture products. Each black dot represents an observation of CPUE from a province in a given year, paired with the observed intensity and extent of aquaculture type in that province and year. Pink indicates positive deviations from the mean observed CPUE; blue indicates negative deviations. The extent of aquaculture was scaled within a province such that ‘0’ represents the average area devoted to a given type of aquaculture over time within a province.

The variability in China’s province-level CPUE in fisheries (a proxy for population size) as a function of the area devoted to cultivating a given type of seafood in China’s provinces can be explained using GAMs with varying degrees of accuracy (31–92% of deviance explained; Fig 10 & Table 2). Significant positive relationships between shellfish and algae culture and total CPUE were observed for 5 of 7 and 4 of 4 provinces, respectively (indicated by relationships that are in color in Fig 9; p-values in Table 2). However, the relationships between total CPUE and area of crustacean culture were mixed with 3 of 6 provinces showing a significant negative relationship (Fig 9 and Table 2). These trends can also be seen in a national level analysis that incorporates intensity of mariculture as an additional axis (Fig 11). Each of the two-dimensional smooths for the different mariculture products were significant in the regression, which explained 56% of the deviance in total CPUE. More intense and more expansive algae operations were generally associated with higher total fisheries CPUE. More intense fish cultivation was related to lower fisheries CPUE and the impact of extent was dependent upon intensity. More intense crustacean farms of a given size were associated with significantly lower fisheries CPUE, with the exception of a few observations of high fisheries CPUE and intense cultivation. Increased intensity of shellfish mariculture was generally associated with significantly higher fisheries CPUE, but increases in extent were associated with lower CPUE. In general, mariculture operations are nearshore and much of China’s catch is obtained off-shore. Even so, negative relationships between mariculture and fisheries may be related to the destruction of nursery grounds of fished species and positive relationships may be a result of farms providing de facto protected areas for juveniles or contributions to water quality.

**Table 2:** Significance of the relationship between province level total CPUE and area devoted to aquaculture or stock enhancement identified by GAMs. P-values are reported here (p < = 0.05 shaded). See Fig 9 for the shapes of the estimated relationships. Deviance explained is in proportion and ranges from 0 to 1.

		Area devoted to aquaculture				Stock enhancement		
		Crustacean	Fish	Shellfish	Algae	Fish	Crustacean	Deviance explained
Province	Liaoning	0.150	0.082	0.000	0.003			0.640
	Hebei	0.001	0.000	0.025		0.390	0.035	0.777
	Tianjin	0.015	0.712			0.000	0.288	0.620
	Shandong	0.153	0.064	0.000	0.020	0.040	0.999	0.650
	Jiangsu	0.369	0.040	0.001	0.929			0.321
	Zhejiang	0.000	0.054	0.012	0.000	0.745	0.016	0.682
	Fujian	0.793	0.000	0.000	0.000			0.831
	Guangdong	0.000	0.000	0.064	0.073	1.000	0.487	0.908
	Hainan	0.000	0.000	0.683	0.723	0.579	0.089	0.917
	Guangxi	0.000	0.195	0.000		0.006	0.581	0.908

China also enhances harvested populations by adding fry reared in captivity to the sea (beginning in the mid-1980s) and constructing artificial reefs. China added 7.8 billion fish larvae to their oceans in 2016 and has supported large-scale artificial reef building since 1979 [5]. The efficacy of each of these techniques varies [25, 26, 27]. In our province-level analysis, stock enhancement appears to be significantly related to total fisheries CPUE in 5 of 7 provinces, however the relationship is not always linear or positive (Fig 9 and Table 2). Targeted analyses for CPUEs of the specific species under enhancement would be a more useful test of the efficacy of enhancement, but those enhancement data are not reported to species level in the Chinese Yearbooks. Time series for artificial reef area by province were not available to test their influence.

## Discussion

China’s marine seafood production strategies provide one of the world’s largest empirical examples of the predicted tradeoff between total ecosystem production and preservation of ecosystem structure. China produces more seafood than any other country in the world (by far) by maintaining the most powerful fishing fleets and implementing large-scale aquaculture operations. We showed that many of the time series of seafood production over time by province and product type increase nearly linearly with effort, but there are exceptions. The species or groups with declining catches are often larger, later maturing species, which aligns with the ecological expectations of the outcomes intense exploitation. We also demonstrated associations between mariculture and fisheries CPUE for which hypotheses could be developed to test. For example, increasing intensity and extent of algae farms are associated with higher total fisheries CPUE, which could be the result of algae farms serving as de facto marine protected areas and increasing available forage. The relationships explored here between production, effort, product types, and production modes are intended to give an overarching picture of the seafood landscape in China and should not be interpreted as causal. Each of the identified relationships are worth closer inspection and we encourage further analysis.

### Data controversy

Our analyses were completed primarily with the reported Chinese fishery data have been questioned in the scientific literature (and aquaculture data to a lesser extent) [28,29]. An oft-heard narrative is that China’s high fisheries catches are an artifact of over-reporting, supported by the assertion that the reported catches outpace primary production [29]. Watson and Pauly [28] first cast doubt on China’s catch statistics when they developed a linear regression to predict catch by country given environmental variables (e.g. depth, primary production, and ice cover). Fishing effort was not included in that model because catches were assumed to be close to their maximum sustainable yield (MSY). China’s catches were deemed misreported based on the fact that China’s catches appeared to be an outlier in the analysis.

There are several issues with the assumptions of this analysis. Costello et al. [30] found that catches for a large fraction of global fisheries were not at levels close to MSY when the original 2001 analysis was performed. The concept of MSY is also highly dependent on the scale at which analyses are done. Other studies have suggested the global MSY could be double current yields, but would come at the cost of larger predatory fish [31,32], as has likely happened in China [33]. Finally, and possibly most importantly, China’s fishing fleets are the most powerful in the world (S1 Fig) and expanded exponentially in the late 1990s around the time when the analysis was published. If a relationship exists between yield and effort (and at the ecosystem level the literature suggests it should be asymptotic for a range of selectivities; [34, 35]), the ‘China effect’ noted by Watson and Pauly could very well reflect the massive increase in Chinese fishing effort, not over-reporting. In spite of fundamental issues with their analysis, the declaration of inaccurate fisheries reporting in China has dominated the conversation around Chinese fisheries to this day.

**Fig 12:**
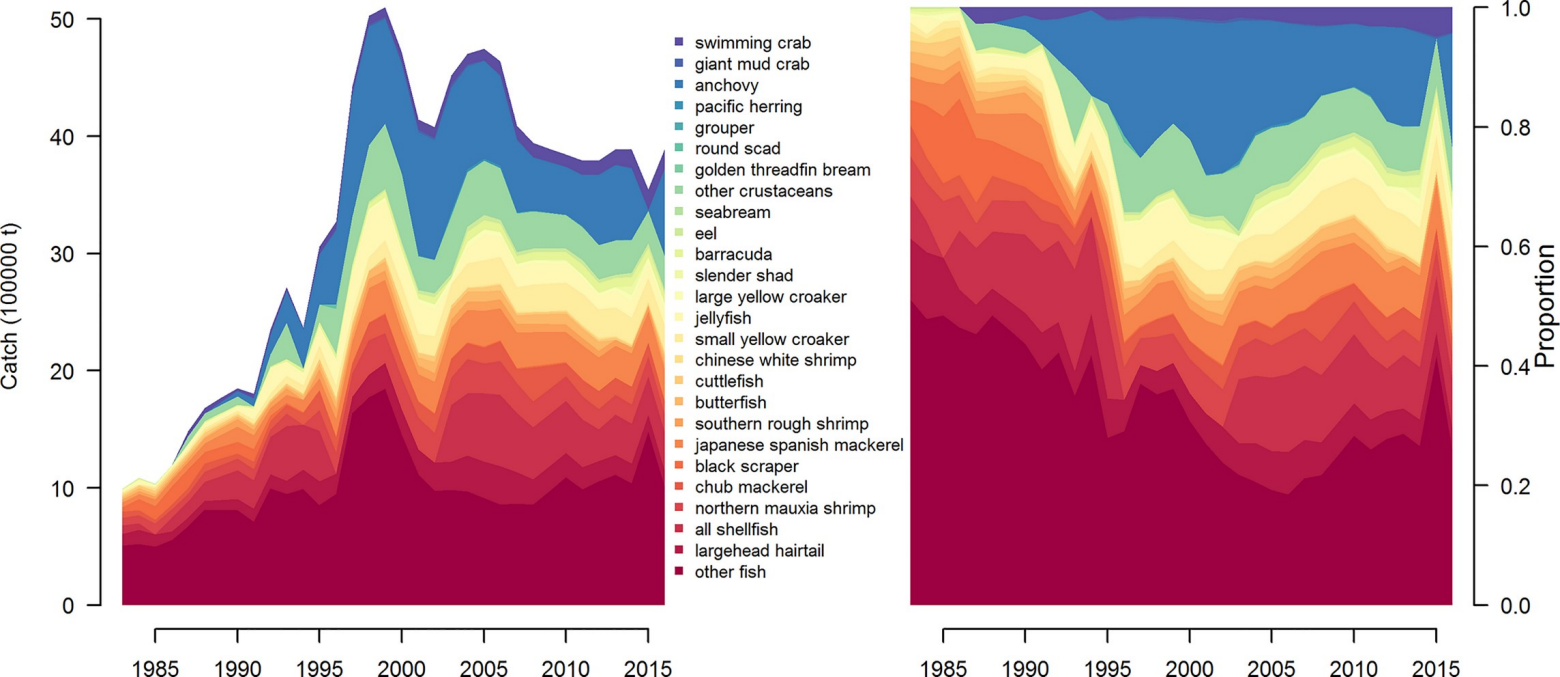
**Time series of catch by species/group in the Yellow Sea and Bohai (left) and the proportion of those species within the catch (right).** Legend is ordered (increasing from the top) by the proportion of species/group in the catch in the first year of the analysis (1986).

An alternative explanation for China’s high reported catches involves changes in trophic structure as a result of intense fishing [33]. Large biomasses of small fish are an expected by-product of ‘fishing down the food web’ [9]. ‘Predatory release’, (in which populations of smaller fish increase after the removal of their predators) has been documented in marine systems around the world [36], but China’s seas appear to be the largest example of this globally. Furthermore, changes observed in China’s catch composition (which should be illustrative of the ecosystem composition because the gear types are indiscriminate) has changed from longer-lived, slower growing species to shorter-lived, faster growing species [37] (Fig 12), which is a predicted outcome of indiscriminate, intense fishing.

The stated goal of published reconstructions of Chinese fisheries catches based on Watson and Pauly’s original analyses is to “adjust the officially reported marine catches of China to a level where they become compatible with reconstructed (rather than official) catches from waters similar to China’s” [38]. This was accomplished by applying a linear regression to estimate the ratio of the total catch reported to the FAO to the ‘corrected’ catch from Watson and Pauly [28]. Then, this regression was used to extrapolate the trend over time in the ratio of reported to ‘corrected’ catch and applied to China’s reported catch. The general result of the reconstructions was a scaling down of reported fishery catches from China (Fig 5). It is somewhat difficult to directly compare the SAU data and the Chinese Fisheries Statistics Yearbook because, although the Chinese only report statistics for 25 species or groups by province, the SAU data report catches for 228 species or groups for the entire country generated from a somewhat opaque algorithm. However, for several of the large commercial species the trends in the time series are roughly preserved (Fig 5). Fisheries ‘status’ (i.e. the state of the fishery with respect to optimal fishing effort and biomass levels) is an oft-used indicator of fishery performance. Inferences drawn on the ‘status’ of resources will remain unbiased even if the magnitude of catches are biased, provided the trends in the data are correct [39]. Based on observed relationships between catch and effort, the inferred levels of optimal effort reported in this analysis are similar regardless of whether the SAU data or the Chinese data are used (Fig 3).

Despite China’s attempts to gather comprehensive fishery catch statistics, potential issues remain with the reported statistics. The world’s largest fisheries catch (China’s) is achieved by the world’s most powerful fleet; policing and observing catch from the entire fleet is a large and difficult task. Fish tickets are a common form of data collection for fisheries globally, but relying on fish tickets also opens the potential for unreported deliveries or discards. For small-scale fisheries in China, fishers tend to record only the value of their catch and the enumerators then calculate the volume in kilograms of the catch from the average price of a given fish. Given the scale of medium to large-scale fisheries, it would be costly to verify the mandatory catch reports via observers in the way that is sometimes done in other countries with smaller fleets. The spatial extent of the fishing of a provincial fleet is also not indicated in the Fishery Yearbooks. We assume that the catches reported by a given province represent the fishing grounds around a given province (which have expanded over time as the fleets have grown). This assumption is likely reasonable given economic constraints, but it is possible that a fraction of the catch reported in a province comes from elsewhere.

Another issue potentially confounding catch reporting is the implementation of a ‘zero growth’ policy implemented in the early 2000s [40] under which the government declared that catches would not increase beyond the then-current levels. Watson and Pauly [28] referred to the zero growth policy as a ‘face saving’ policy implemented in response to accusations of over-reporting. However, it could also have been a response to observed declines in CPUE with the goal of curbing massive fleet growth. The Chinese government has made downward revisions of catch reports in the past, and there are potential issues in China’s fishery data. However, we believe the official fishery statistics from China (rather than catch reconstructions) are appropriate metrics to use in our analysis, given sometimes opaque and questionable assumptions made in reconstructions outlined above and potential alternative explanations for China’s large fisheries catches. Further, based on the limited comparisons that are feasible between the SAU data and Chinese data, input-based management advice derived from these analyses would be very similar.

### Implications for management: Costs, benefits, risks, and markets

Three benefits of intense capture and cultivation in China are apparent from our analysis: high yields, high employment, and low management costs. High capture yields appear to be derived from restructuring of ecosystems and supplementation from distant waters; high cultivation yields have followed ubiquitous, dense mariculture farms. The scale of China’s seafood production necessitates a large workforce with minimal education requirements—an estimated 5.7 million workers in 2016 were involved with the production of seafood in China [5]. Mangin et al. [41] estimated the cost of single-species management averages US$489 per metric ton of catch. Based on these estimates, implementing fisheries management similar to developed countries would cost China at least US$7 billion. This is likely an underestimate, given these costs were derived primarily from countries with fleets an order of magnitude smaller than China’s and one of the most laborious tasks in management is accounting for catch coming off of each boat.

Still, many ecological and economic costs accompany the benefits of intense cultivation and exploitation. Overfishing (as defined in the single species paradigm) has occurred on an unprecedented scale in China’s waters—the majority of fish in the catch are only a year old, community composition has shifted to lower value species over time [42] (Fig 3), and fish mature earlier and grow to a smaller maximum size more quickly than 30 years ago [43]. Shark and marine mammal populations have been impacted [44] and larger individuals of commercial species are mostly absent from Chinese waters [43]. Many more species are caught than are enumerated in the Fisheries Yearbooks—declines and disappearances of some species are likely masked by their inclusion in the ‘other fish’ category.

The low observed CPUEs in China require high effort to produce high catches; effort is maintained through massive fuel subsidies ($6.3 billion USD in 2013; [45]). In response to this investment, China’s fishing fleets have expanded, and demand for seafood has driven fishers to waters outside of China’s. A potentially positive outcome of Chinese fleets fishing in foreign waters is that countries may derive benefit from their resources without building their own fleets (e.g., by charging for permits to fish within their EEZ). However, if a regulatory infrastructure does not exist in those countries to govern the details of these arrangements, the potentially negative impacts on ecosystems and local markets may be large. China’s fishing methods may decrease the size and/or number of the species traditionally caught by local fishermen because of differences in fishing methods. Further, it is not clear if the flow of potential benefits would reach the impacted stakeholders. The details of the agreements between Chinese fishers and the host countries are not usually public, so it is difficult to evaluate the impacts of China’s distant water fleets or understand the consequences for stakeholders. Mallory [46] reported that China largely appears to follow international guidelines on sustainable fisheries, but suggested that improvements could be made in terms of the use of flags of convenience and illegal, unreported and unregulated fishing that could improve international relationships and transparency of the impacts of distant water fishing fleets.

The use of wild fish for feed by China’s agriculture and aquaculture sector is increasing as a result of growth, the rising share of fed species in production, and the intensification practices for non-obligate carnivores, like carp [47]. Not surprisingly, China is the world’s largest importer of fish meal, as domestic supply is limited due to declines in targeted reduction fisheries such as Japanese anchovy. The depletion of “trash” fisheries is a growing concern, with China reportedly using ~3.4 million metric tonnes (mmt) each year for feed in aquaculture [48]. Although promising alternative feeds are emerging to replace fish meal, the use of fish meal remains profitable in China [49].

Health and safety concerns have been raised as aquaculture develops in China. Antibiotics are used to prevent disease in aquaculture and are washed into China’s coastal waters [50]. Excessive antibiotic use can lead to resistant pathogens that threaten human health [51] and has been linked to higher prevalence of antibiotic-resistant bacteria in humans that live near aquaculture in China [52]. Mariculture products and the surrounding environment can also accumulate heavy metals and other toxic compounds originating from farm structures (paint, antifoulants), pigments in feed, disinfectants, and chemotherapeutants [53]. Concerns over the presence of banned antibiotic residues and other harmful compounds have caused widespread bans on imports of Chinese aquaculture products in the past, and controls have been introduced to attempt to prevent these problem [54]. However, published data on whether or not measures aimed at controlling their use have been successful are difficult to find [55, 56].

In addition to these economic and ecological costs, there are potential risks associated with modifying ecosystem structure through intense exploitation and cultivation. Conservation of natural ecosystem structure and diversity is often linked to resilience in the face of disturbance both in theory and empirically [57]. Many cultivated species in China are non-native and accidental releases or hybridizations can adversely impact natural ecosystems [58, 59]. Diverse ‘portfolios’ of exploited stocks are thought to provide insurance against environmental change, such that, regardless of conditions, a stock will be present that can thrive [60]. Similarly, loss of ‘age diversity’ through truncation of the age structure within a population of a single species may have a host of negative influences (e.g. reducing productivity, trait diversity, and community stability; [61]). However, some systems do not follow the trend of requiring diversity to support resilience [62] and marine systems may operate counter to the dynamics predicted by ecosystem theory due, in part, to the generalist diets of many fish [63].

Climate change is another potential risk to seafood production using China’s strategy. *In situ* mariculture production is largely determined by ocean conditions and is expected to be affected by climate change, but the effects will depend on species and location. Where ocean temperatures are currently sub-optimal for growth, productivity of some cultured species is expected to rise (e.g. cobia, *Rachycentron canadum*; [64]). In contrast, shellfish mariculture is vulnerable to ocean acidification, which is a threat to China’s seafood production given the importance of shellfish in China’s portfolio [65]. Integrated multi-trophic aquaculture systems may mitigate some of the risk associated with environmental change [17], but more research into this subject would be useful. Beyond aquaculture, there is disagreement in the literature on the impact of climate change on China’s fisheries. Cheung et al. [66] predicted that China will be among the countries to see the biggest losses in fisheries catch due to climate change; Allison et al. [67] did not include China in their list of countries most vulnerable to impacts of climate change.

### Moving forward

Intense fishing (and the associated costs, benefits, and risks) currently appears to be a viable strategy in China because a domestic market exists for nearly all species and sizes of fish, unlike many other seafood markets around the world. Two forces drive this market: culinary tastes and aquaculture feed. Culturally, small fish cooked whole are a part of the culinary tradition. Fish too small for human consumption can readily be used as feed for farmed fish. As a result of these two drivers, discarding does not exist in China as it does in other parts of the world (particularly those implementing single species management). It is not certain, however, that these market pressures will remain constant in the future. In general, a relationship between environmental quality and per capita income exists—as a country begins to develops economically, environmental quality declines. However, once per capita income is sufficiently high, environmental quality improves, presumably because the demand for a clean environment (and the means to pay for it) increases (the “Kuznets curve” [68]). China is approaching the per capita income level at which this shift often occurs and pressure for improved environmental quality is being observed in other sectors (e.g. water and air quality [69]). It remains to be seen if increases in income will also increase demand for ‘natural’ marine ecosystems (i.e. those with species and structure similar to those 50 years ago) and shifts in culinary tastes.

A major focus of China’s most recent 5-year plan is to improve the ecological condition of their oceans and strides have been made towards accomplishing this goal [40]. For example, in fisheries, pilot projects aimed at improving management are currently underway in coastal provinces and the length of the closed season was extended a month in 2017, from three months to approximately four [4]. In 2018, inshore mariculture was limited by China’s government, and mariculture is now encouraged to develop in offshore waters. China’s goals for 2020 are to decrease catches from wild capture fisheries to 10 million tons, eliminate 40% of fisheries subsidies, and reduce the fishing fleet by 20,000 vessels and 1.5 million kilowatts [4]. Reducing fisheries subsidies will likely make a fraction of the fishing effort expended by Chinese fleet unprofitable (particularly those on the high seas, [70]) and help achieve these goals. Redistributing these subsidies to other efforts (e.g. monitoring, enforcement, and reeducation) could improve the social and ecological outcomes of ocean management [71]. The ecological and economic impacts of decreasing catch and fleet capacity will largely depend on how these management reforms are realized. One of the key choices is between maintaining indiscriminate, multispecies fisheries and attempting to implement single-species, quota-based management.

Implementing single-species management may have socially harmful consequences due to differences in fishing methods, ecosystem structure, and markets between the developed and developing world. Szuwalski et al. [33] demonstrated that implementing single-species fisheries management could halve the amount of catch coming from the East China Sea. A growing body of evidence demonstrating the ‘cultivation effect’ due to predatory release in marine fisheries exists [36, 72], by which changes in trophic structure resulting from fishing can increase catches. Single-species management could reverse this cultivation effect by rebuilding populations of predators and ultimately decreasing catches. In general, implementation of fisheries management in developed countries has coincided with decreases in total catches, whereas catches from countries with less developed management systems continue to increase [73]. Still, management reform has increased CPUE in developed countries, whereas CPUE in developing countries has continually declined [74], which can affect the profitability of fisheries. Reducing catches in China could increase China’s reliance on imported seafood [1] and presumably increase prices for some seafood products (not just in China, but around the world). Single-species management could also reduce the number of jobs available to people working in fisheries in China and introduce perverse incentives like discarding and high-grading not currently prevalent in Chinese fisheries.

China plans to decrease total seafood production to 66 million tons by 2020 from 69 million tons in 2017 [4], which, coupled with the planned 5 million ton decreases in wild capture production, translates to needed increases in aquaculture production. This goal will continue the trend of shifting the burden of seafood production from captured to cultured seafood in China. Increased reliance on cultured seafood is potentially rational because farmed populations can be controlled more closely than wild populations, so it may be possible to mitigate some of the risks and costs described above. Still, even in polycultures, the stability of the portfolio effect provided by natural ecosystems is removed.

In addition to differences in species composition and resilience, the management challenges for aquaculture and fisheries are different. Two systems of monitoring aquaculture appear to exist in China: one for international markets (primarily the U.S, Japan, and the E.U.) and one for the domestic market (accounting for >85% of China’s total aquaculture production [54]). The system for international markets is regulated more closely than the domestic market, given international standards, and adopting these for all cultured seafood in China would be costly. For example, Norway exports 95% of its aquaculture product and the E.U. (which has stringent controls on import of aquaculture products relative to the world) is a primary consumer. Norway spent ~US$137 million dollars to ensure the safety and quality of 600 thousand tons of product in 2005 [75]. If China were to implement a similar system for all of its aquaculture farms, the cost would be more than US$10 billion. Costs could be higher than this due to the prevalence of small-scale live fish markets and the difference in geographic scales between Norway and China.

The pressures China faces while developing sustainable strategies for seafood production are emblematic of countries with minimal management of fisheries (which produce nearly half of the world’s wild-capture catch; Hilborn et al., [76]) and large aquaculture operations. China’s experiments in seafood production have provided a large-scale learning opportunity on the outcomes of intense exploitation and cultivation aimed at increasing volume of seafood produced. China has indicated that their future plans for seafood production will depart from business as usual and focus more on quality, rather than just quantity. It is not immediately clear that importing the developed world’s fisheries management frameworks can simultaneously maintain seafood production and achieve China’s ecological goals. Further, no examples exist globally on which to draw for aquaculture reform given the advanced state of Chinese aquaculture. So, China may have to develop novel management methods to address the challenges associated with producing marine seafood at this scale, including, but not limited to, improving methods for monitoring and enforcement for large fishing fleets and aquaculture systems, workforce reeducation, and developing theory for alternative management strategies for multispecies fisheries. Harmonization and centralization of the available fisheries, aquaculture, and environmental data would facilitate a data-driven approach to reform, as would establishing a centralized database of scientific data related to marine ecosystems. Whatever strategy and tactics for producing seafood are developed in China, our analysis suggests that the interactions among and within cultured and wild systems will need to be carefully considered in reform efforts. The successful development of methods to address the challenges China faces in marine resource management described here would be exceedingly useful for many other countries in the region facing pressures to simultaneously support ecosystem structure and function and maintain seafood production.

## Supporting information

S1 Fig Totals for marine seafood production by species/groups from 1983–2016 in Zhejiang province. Time series are grouped via hierarchical clustering so that time series with similar ‘shapes’ are together. Colors represent the value of a species in a given year relative to the arithmetic mean for that species/group. Red values are below the mean; blue values are above. Names printed in bold italics are aquaculture production and the number in parentheses is the maximum value for that species/group in 10,000 t.
(DOCX)

S2 Fig Same as S1 Fig, but for Shandong. (DOCX)

S3 Fig Same as S1 Fig, but for Fujian. (DOCX)

S4 Fig Same as S1 Fig, but for Guangdong. (DOCX)

S5 Fig Same as S1 Fig, but for Guangxi. (DOCX)

S6 Fig Same as S1 Fig, but for Hainan. (DOCX)

S7 Fig Same as S1 Fig, but for Hebei. (DOCX)

S8 Fig Same as S1 Fig, but for Jiangsu. (DOCX)

S9 Fig Same as S1 Fig, but for Liaoning. (DOCX)

S10 Fig Same as S1 Fig, but for Tianjin. (DOCX)

S11 Fig Effort clusters by product type (e.g. fishing vs. area of shellfish aquaculture) from 1983–2016 for all provinces. Time series are grouped via hierarchical clustering so that time series with similar ‘shapes’ are together. Colors represent the value of a species in a given year relative to the arithmetic mean for that species/group. Red values are below the mean; blue values are above.
(DOCX)

S12 Fig Hectares devoted to mariculture over time by province with LOESS line overlaid. (DOCX)
