# Supplementary material for: Marine seafood production via intense exploitation and cultivation in China: Costs, benefits, and risks
Source: PLoS One. 2020 Jan 17;15(1):e0227106. doi: 10.1371/journal.pone.0227106 (PMC6968841; doi:10.1371/journal.pone.0227106)
Supplement: S1 Fig — Time series are grouped via hierarchical clustering so that time series with similar ‘shapes’ are together. Colors represent the value of a species in a given year relative to the arithmetic mean for that species/group. Red values are below the mean; blue values are above. Names printed in bold italics are aquaculture production and the number in parentheses is the maximum value for that species/group in 10,000 t. (DOCX) [file pone.0227106.s001.docx]

**Supporting information**


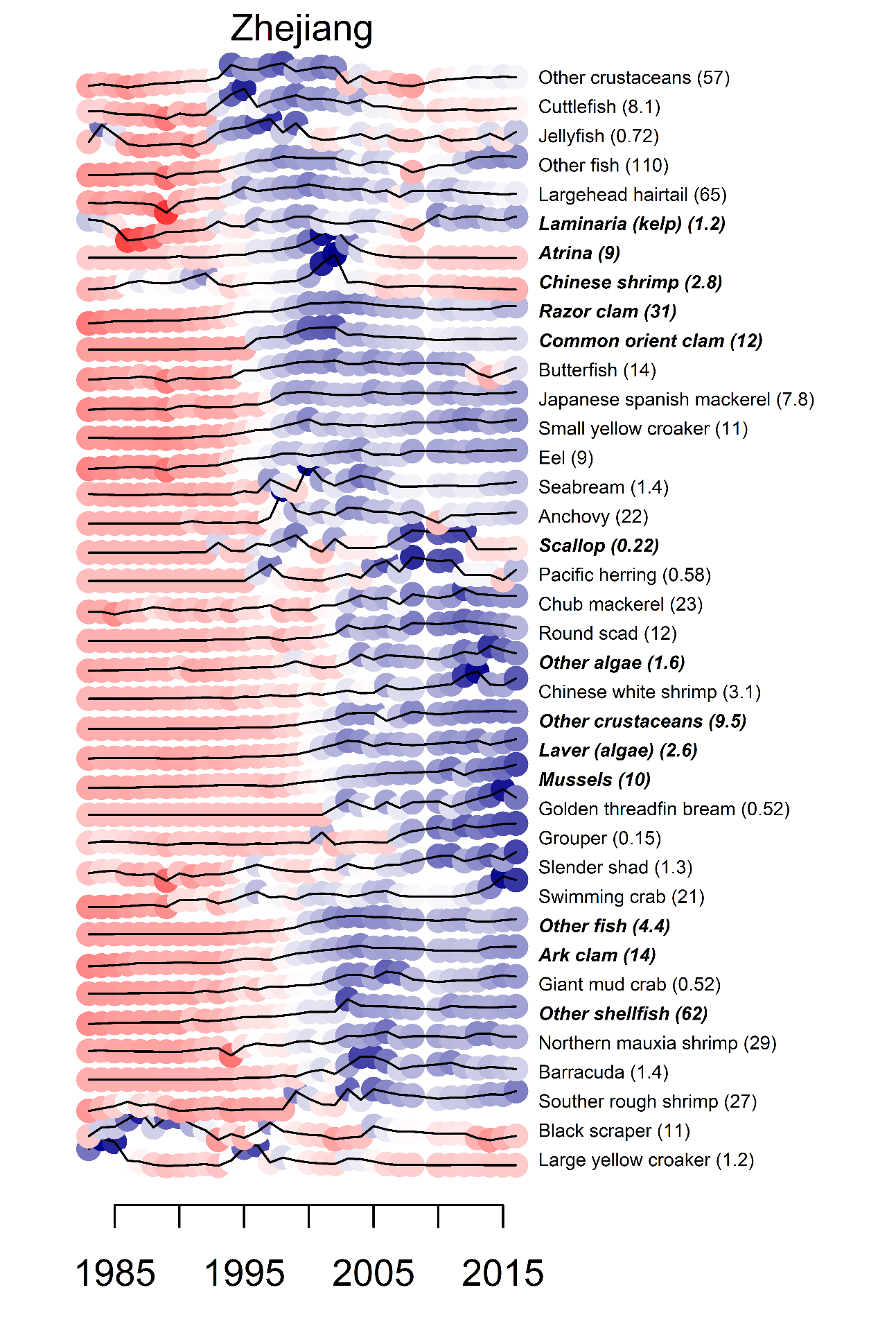


S1 Fig. Totals for marine seafood production by species/groups from 1983-2016 in Zhejiang province. Time series are grouped via hierarchical clustering so that time series with similar ‘shapes’ are together. Colors represent the value of a species in a given year relative to the arithmetic mean for that species/group. Red values are below the mean; blue values are above. Names printed in bold italics are aquaculture production and the number in parentheses is the maximum value for that species/group in 10,000 t.
