# Supplementary figures and images for: Marine seafood production via intense exploitation and cultivation in China: Costs, benefits, and risks
Source: PLoS One. 2020 Jan 17;15(1):e0227106. doi: 10.1371/journal.pone.0227106 (PMC6968841; doi:10.1371/journal.pone.0227106)

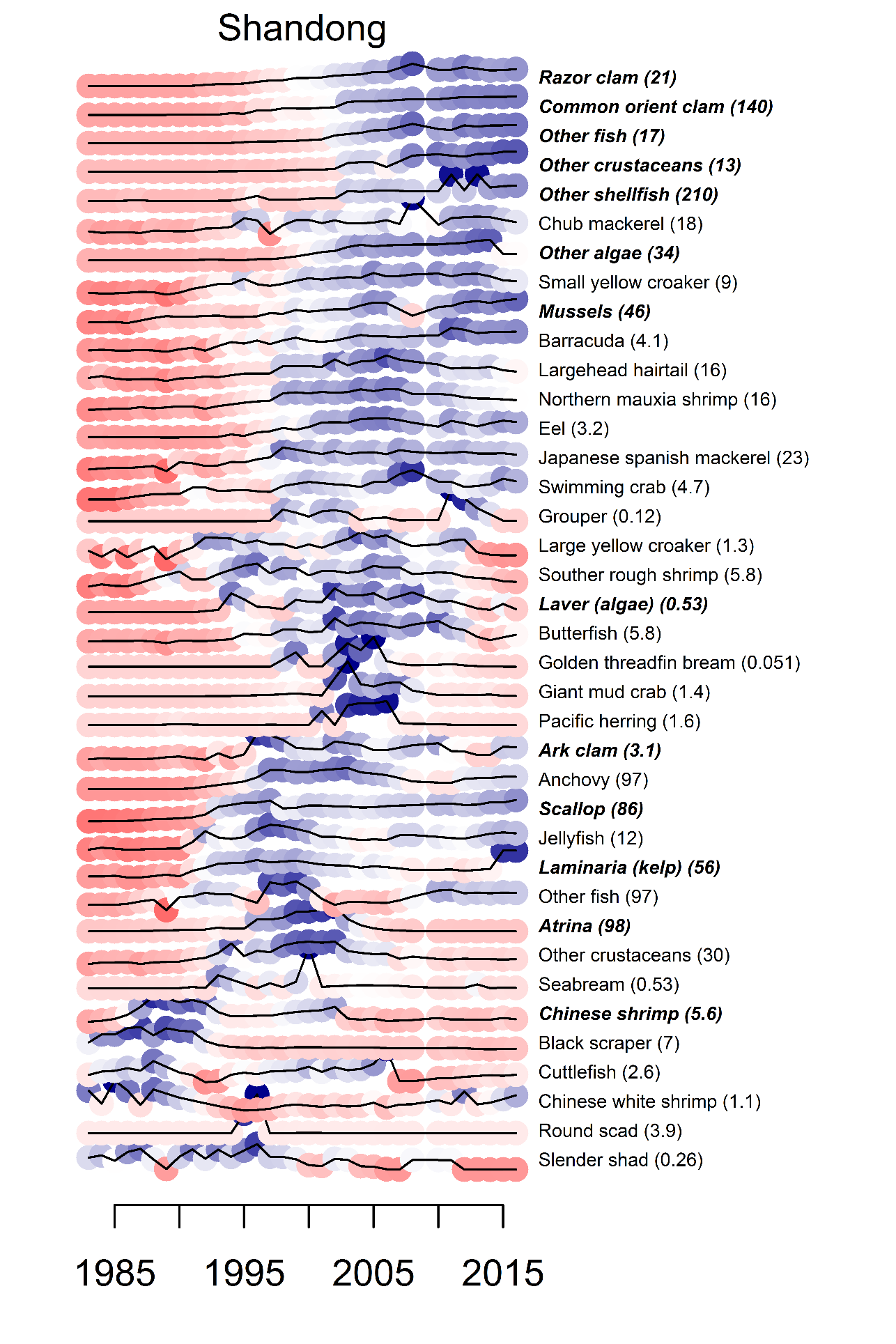


S2 Fig. Same as figure S1, but for Shandong.

Supplement: S2 Fig — (DOCX) [file pone.0227106.s002.docx]

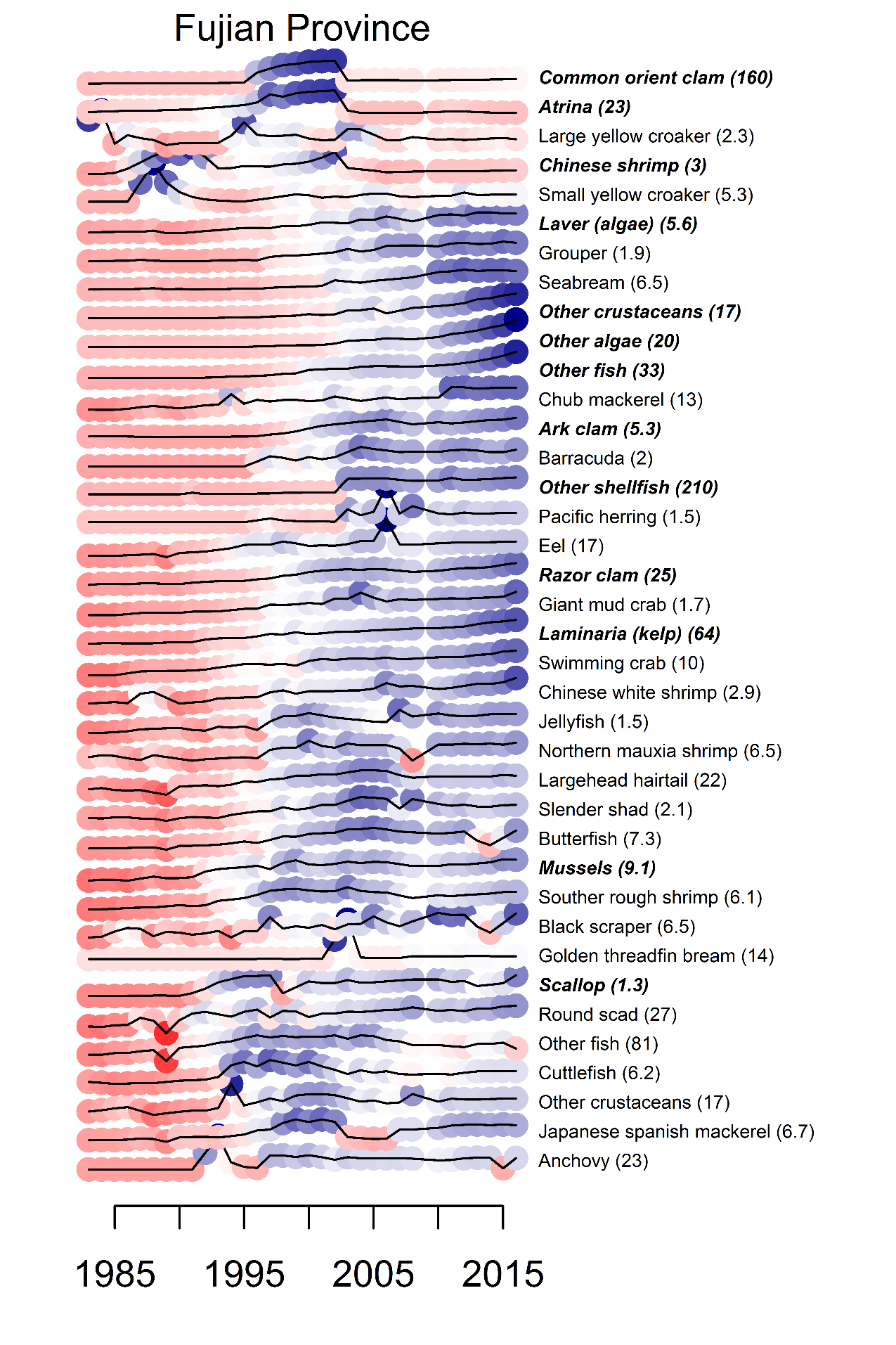


S3 Fig. Same as figure S1, but for Fujian.

Supplement: S3 Fig — (DOCX) [file pone.0227106.s003.docx]

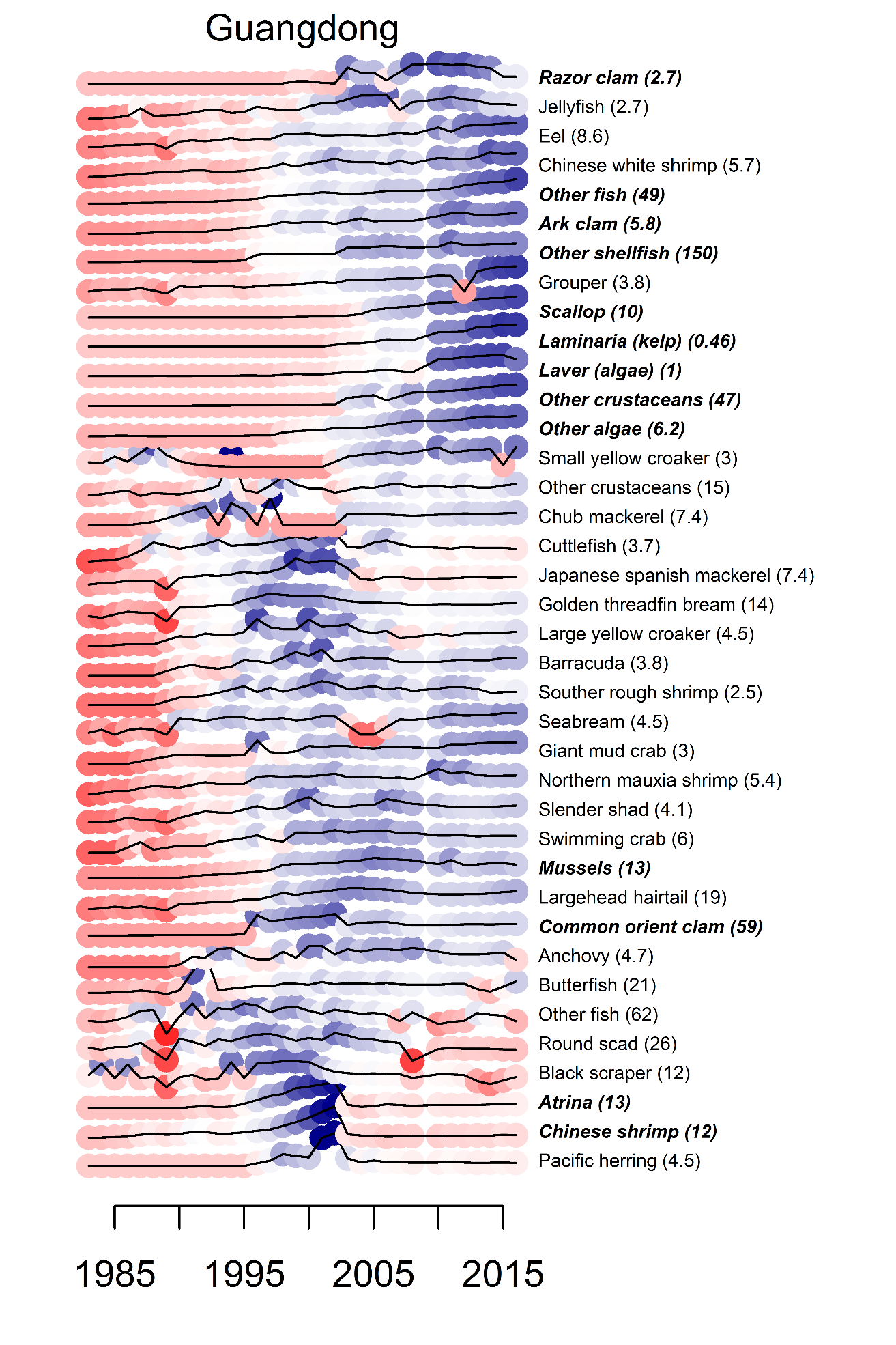


S4 Fig. Same as figure S1, but for Guangdong.

Supplement: S4 Fig — (DOCX) [file pone.0227106.s004.docx]

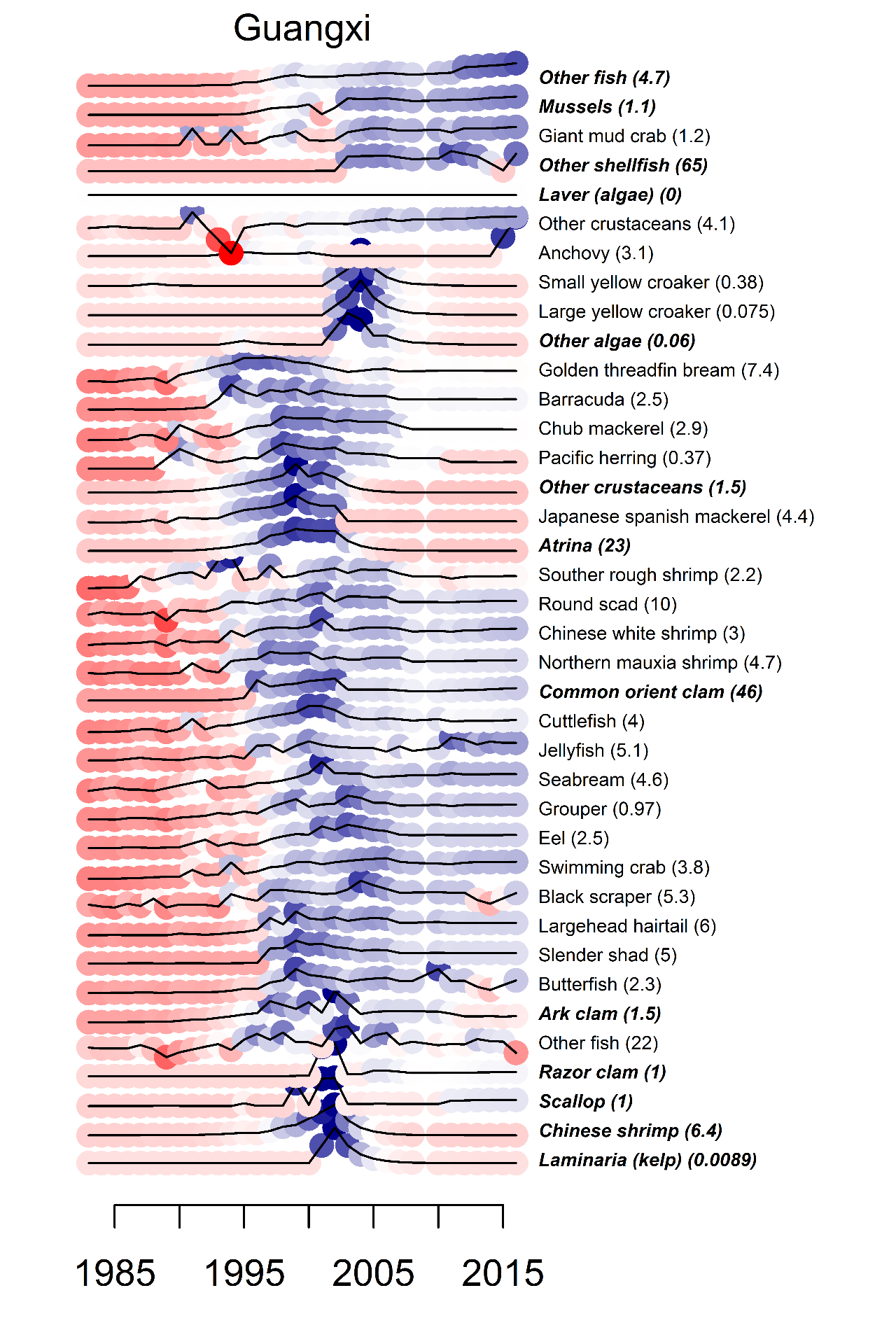


S5 Fig. Same as figure S1, but for Guangxi.

Supplement: S5 Fig — (DOCX) [file pone.0227106.s005.docx]

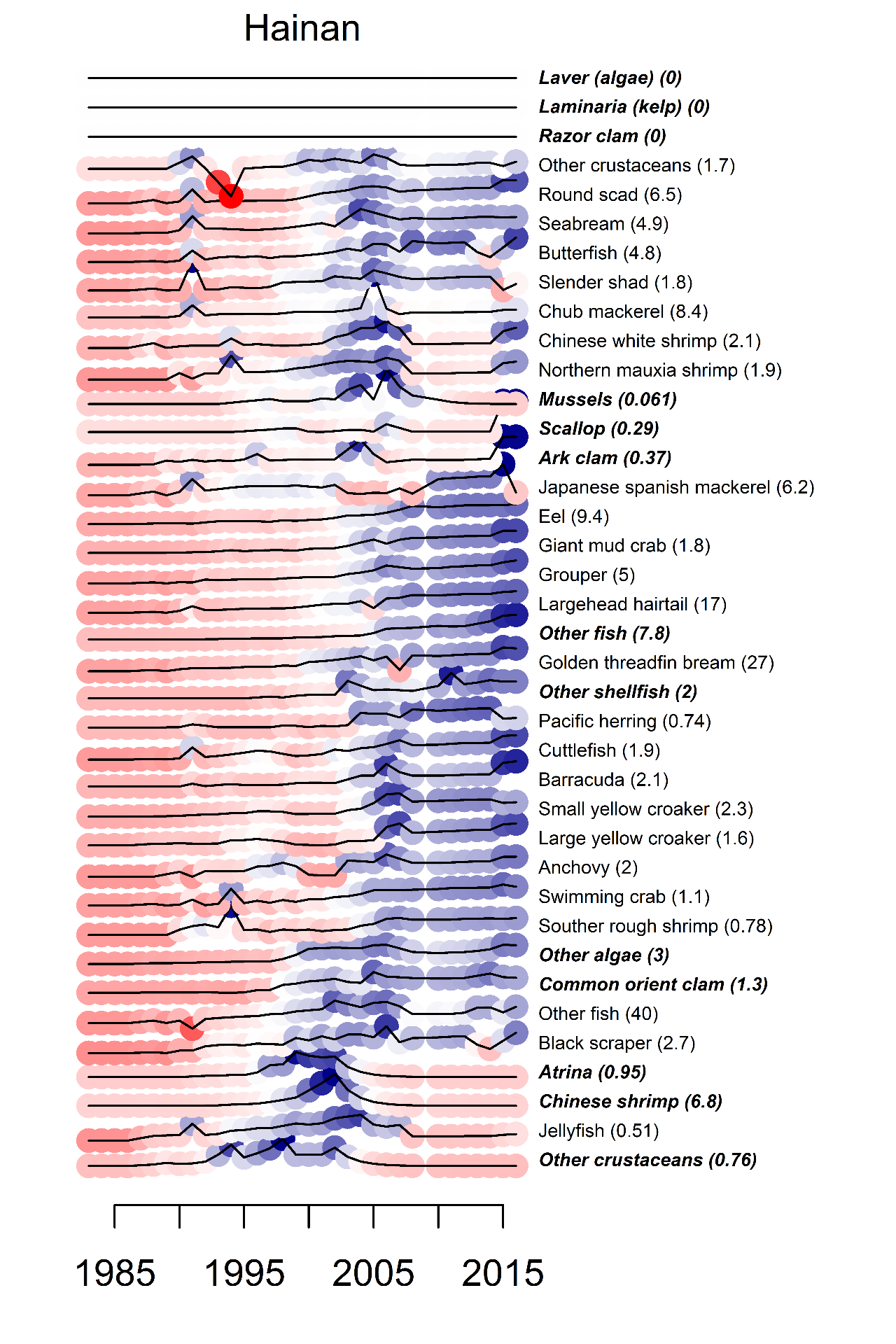


S6 Fig. Same as figure S1, but for Hainan.

Supplement: S6 Fig — (DOCX) [file pone.0227106.s006.docx]

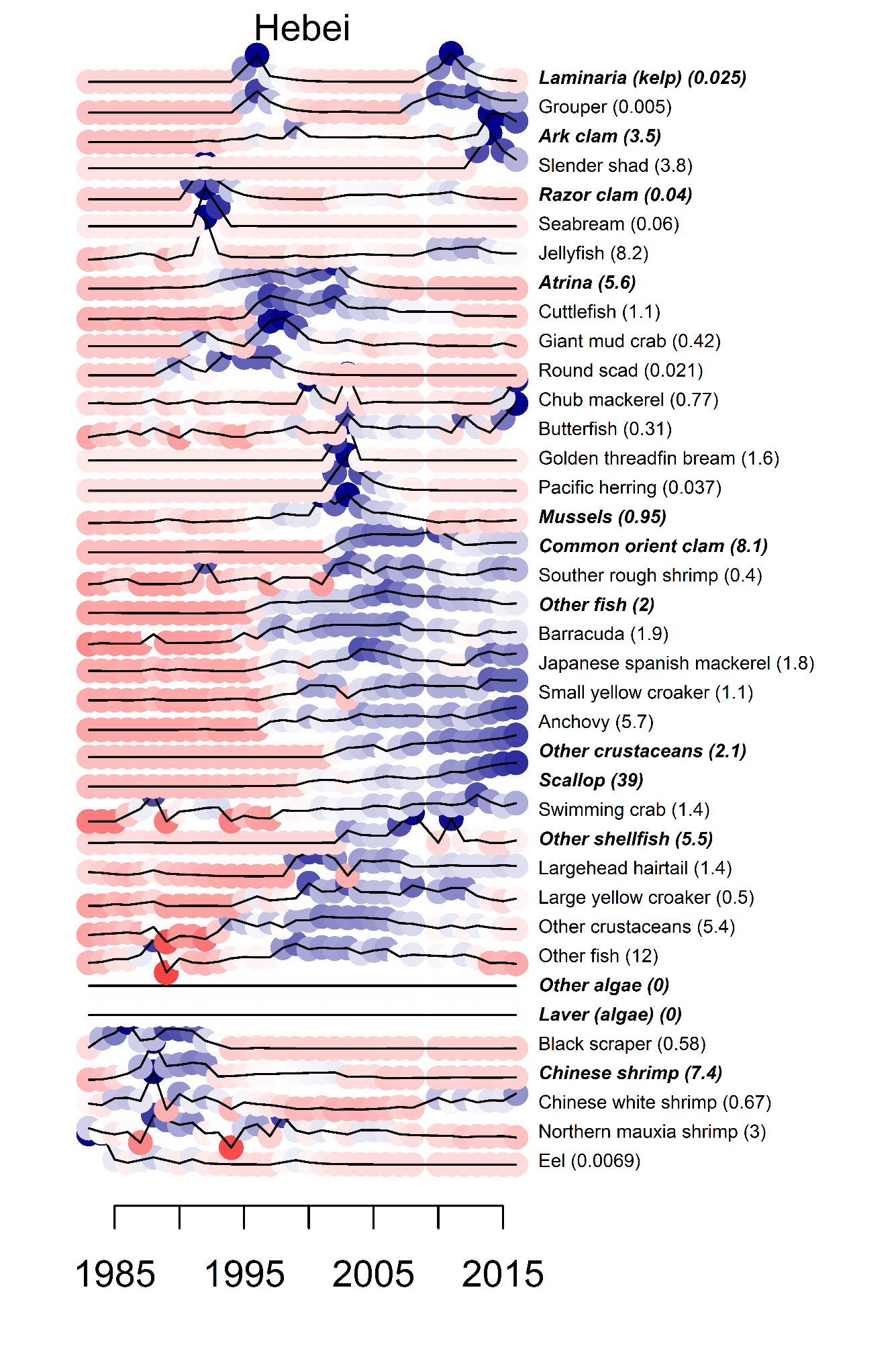


S7 Fig. Same as figure S1, but for Hebei.

Supplement: S7 Fig — (DOCX) [file pone.0227106.s007.docx]

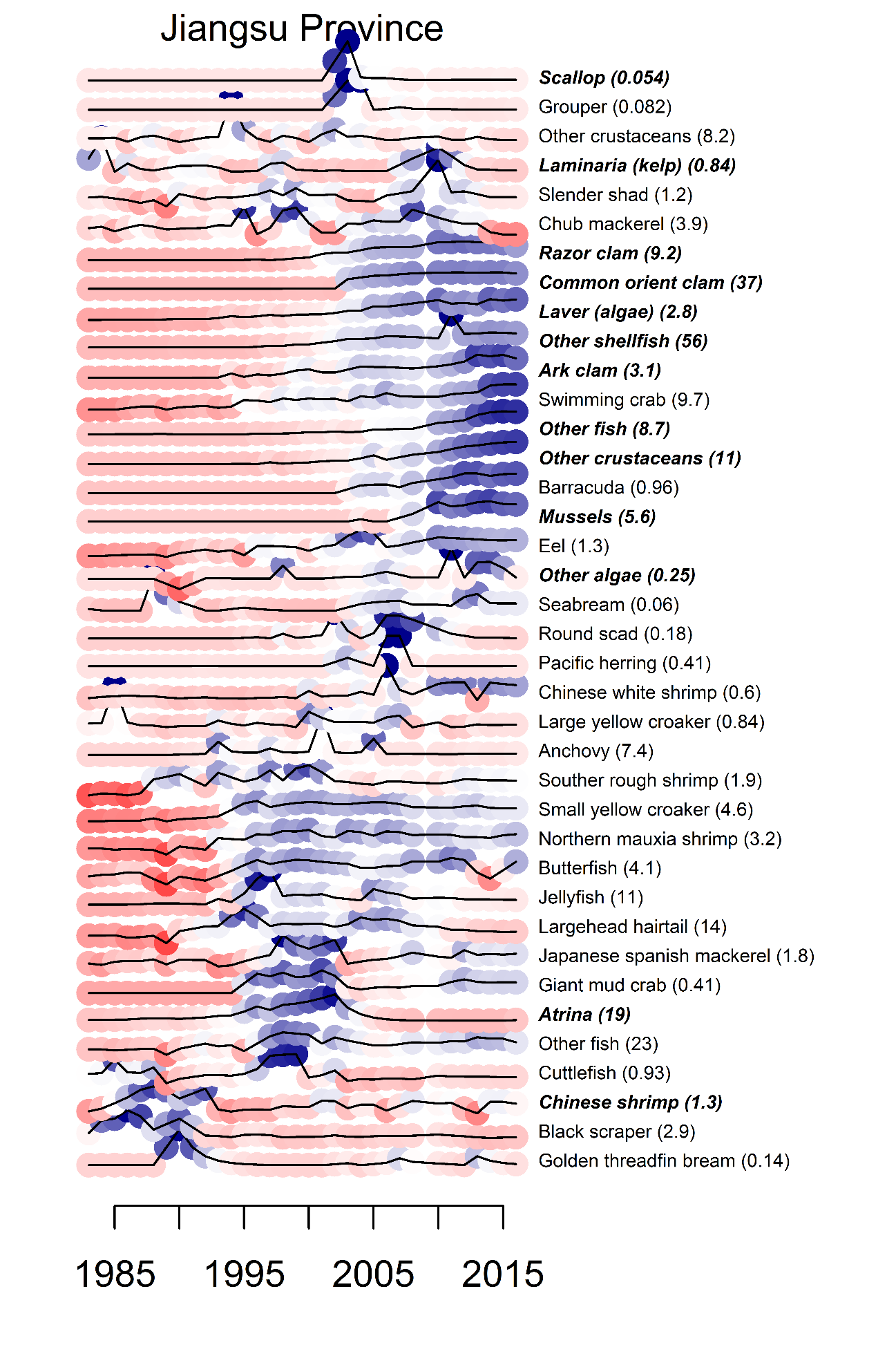


S8 Fig. Same as figure S1, but for Jiangsu.

Supplement: S8 Fig — (DOCX) [file pone.0227106.s008.docx]

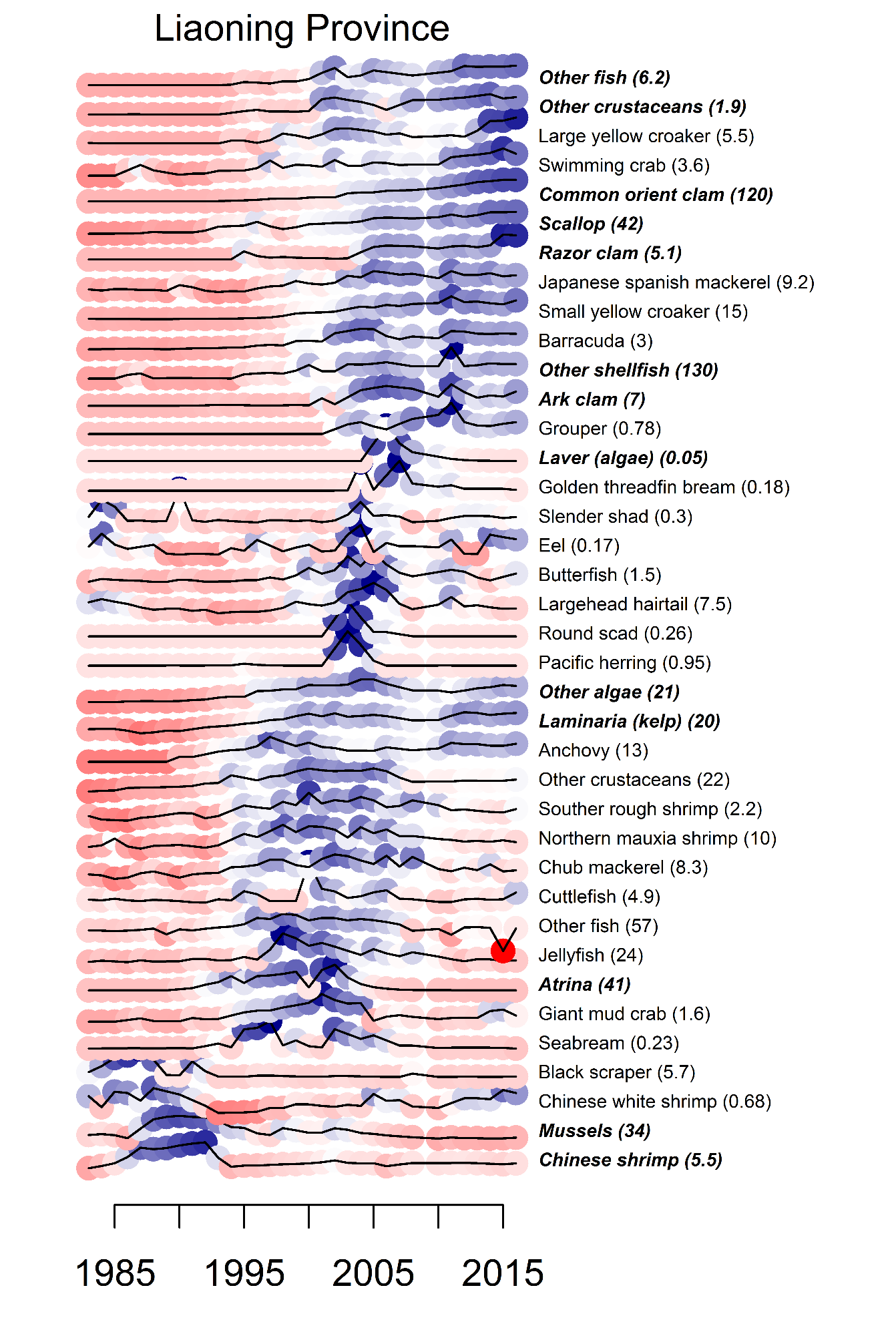


S9 Fig. Same as figure S1, but for Liaoning.

Supplement: S9 Fig — (DOCX) [file pone.0227106.s009.docx]

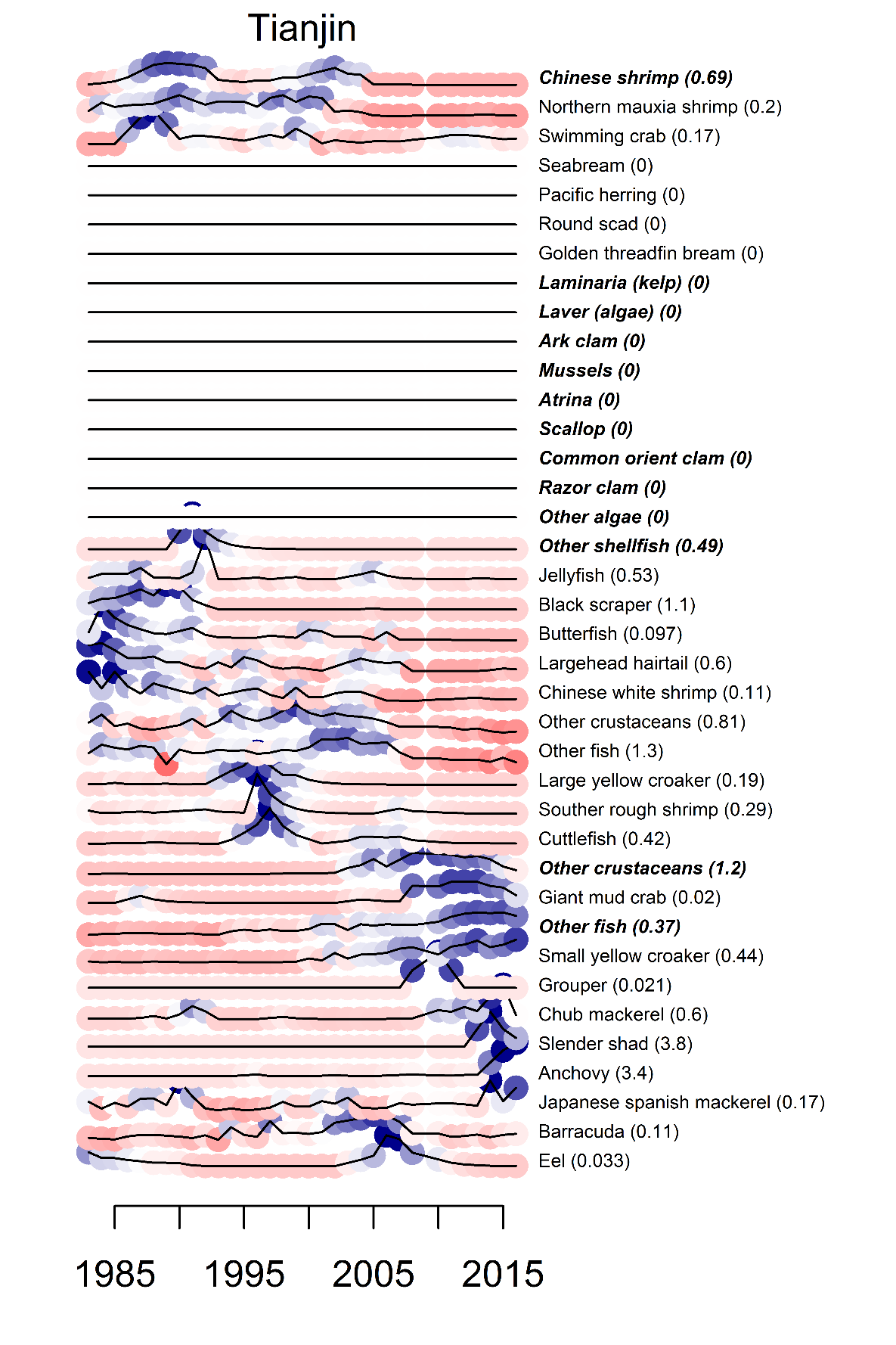


S10 Fig. Same as figure S1, but for Tianjin.

Supplement: S10 Fig — (DOCX) [file pone.0227106.s010.docx]
