# Supplementary material for: Marine seafood production via intense exploitation and cultivation in China: Costs, benefits, and risks
Source: PLoS One. 2020 Jan 17;15(1):e0227106. doi: 10.1371/journal.pone.0227106 (PMC6968841; doi:10.1371/journal.pone.0227106)
Supplement: S11 Fig — Time series are grouped via hierarchical clustering so that time series with similar ‘shapes’ are together. Colors represent the value of a species in a given year relative to the arithmetic mean for that species/group. Red values are below the mean; blue values are above. (DOCX) [file pone.0227106.s011.docx]

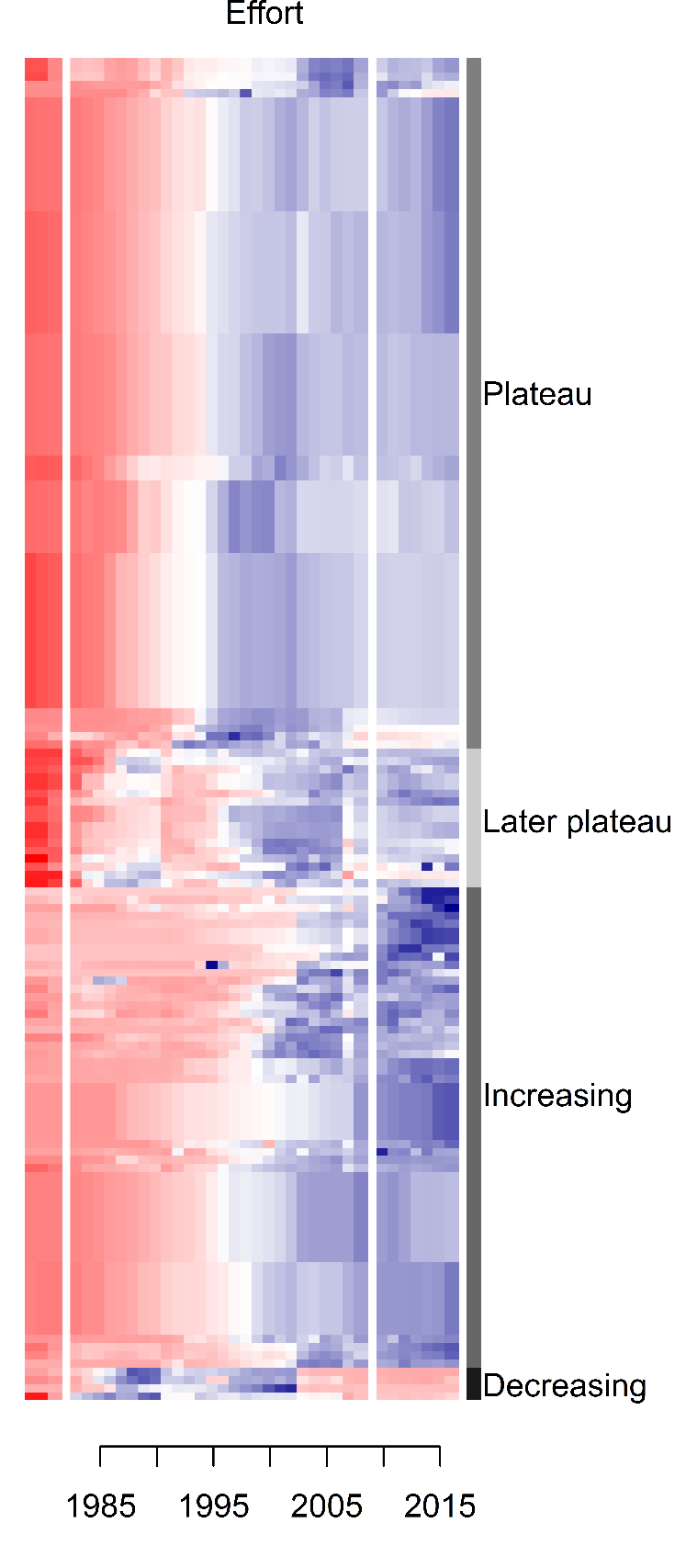


S11 Fig. Effort clusters by product type (e.g. fishing vs. area of shellfish aquaculture) from 1983-2016 for all provinces. Time series are grouped via hierarchical clustering so that time series with similar ‘shapes’ are together. Colors represent the value of a species in a given year relative to the arithmetic mean for that species/group. Red values are below the mean; blue values are above.
