# Supplementary material for: Marine seafood production via intense exploitation and cultivation in China: Costs, benefits, and risks
Source: PLoS One. 2020 Jan 17;15(1):e0227106. doi: 10.1371/journal.pone.0227106 (PMC6968841; doi:10.1371/journal.pone.0227106)
Supplement: S12 Fig — (DOCX) [file pone.0227106.s012.docx]

S12 Fig. Hectares devoted to mariculture over time by province with LOESS line overlaid.
